# Comprehensive evaluation of genome-wide 5-hydroxymethylcytosine profiling approaches in human DNA

**DOI:** 10.1186/s13072-017-0123-7

**Published:** 2017-04-20

**Authors:** Ksenia Skvortsova, Elena Zotenko, Phuc-Loi Luu, Cathryn M. Gould, Shalima S. Nair, Susan J. Clark, Clare Stirzaker

**Affiliations:** 1 Epigenetics Research Laboratory, Genomics and Epigenetics Division, Garvan Institute of Medical Research, 384 Victoria Street, Darlinghurst, Sydney, NSW 2010 Australia; 2 St Vincent’s Clinical School, UNSW Australia, Sydney, NSW 2010 Australia

**Keywords:** DNA methylation, Methylome, Epigenetics, 5-Hydroxymethylation, HM450K Bis/OxBis, hMeDIP-seq

## Abstract

**Background:**

The discovery that 5-methylcytosine (5mC) can be oxidized to 5-hydroxymethylcytosine (5hmC) by the ten-eleven translocation (TET) proteins has prompted wide interest in the potential role of 5hmC in reshaping the mammalian DNA methylation landscape. The gold-standard bisulphite conversion technologies to study DNA methylation do not distinguish between 5mC and 5hmC. However, new approaches to mapping 5hmC genome-wide have advanced rapidly, although it is unclear how the different methods compare in accurately calling 5hmC. In this study, we provide a comparative analysis on brain DNA using three 5hmC genome-wide approaches, namely whole-genome bisulphite/oxidative bisulphite sequencing (WG Bis/OxBis-seq), Infinium HumanMethylation450 BeadChip arrays coupled with oxidative bisulphite (HM450K Bis/OxBis) and antibody-based immunoprecipitation and sequencing of hydroxymethylated DNA (hMeDIP-seq). We also perform loci-specific TET-assisted bisulphite sequencing (TAB-seq) for validation of candidate regions.

**Results:**

We show that whole-genome single-base resolution approaches are advantaged in providing precise 5hmC values but require high sequencing depth to accurately measure 5hmC, as this modification is commonly in low abundance in mammalian cells. HM450K arrays coupled with oxidative bisulphite provide a cost-effective representation of 5hmC distribution, at CpG sites with 5hmC levels >~10%. However, 5hmC analysis is restricted to the genomic location of the probes, which is an important consideration as 5hmC modification is commonly enriched at enhancer elements. Finally, we show that the widely used hMeDIP-seq method provides an efficient genome-wide profile of 5hmC and shows high correlation with WG Bis/OxBis-seq 5hmC distribution in brain DNA. However, in cell line DNA with low levels of 5hmC, hMeDIP-seq-enriched regions are not detected by WG Bis/OxBis or HM450K, either suggesting misinterpretation of 5hmC calls by hMeDIP or lack of sensitivity of the latter methods.

**Conclusions:**

We highlight both the advantages and caveats of three commonly used genome-wide 5hmC profiling technologies and show that interpretation of 5hmC data can be significantly influenced by the sensitivity of methods used, especially as the levels of 5hmC are low and vary in different cell types and different genomic locations.

**Electronic supplementary material:**

The online version of this article (doi:10.1186/s13072-017-0123-7) contains supplementary material, which is available to authorized users.

## Background

DNA cytosine methylation is one of the key epigenetic determinants of mammalian gene expression in normal development [1, 2] and disease [3]. DNA methylation is particularly dynamic during early embryonic development followed by “dynamic homeostasis” of the methylation landscape in normal functioning somatic cells [2]. The discovery that 5-methylcytosine (5mC) can be oxidized to 5-hydroxymethylcytosine (5hmC) by the ten-eleven translocation (TET) proteins has prompted wide interest in the potential roles of 5hmC in reshaping the mammalian DNA methylation landscape during early embryonic development [4, 5], during differentiation towards extra-embryonic lineages [6, 7] and in metabolically active normal adult tissues [8, 9] and disease cells [10]. 5hmC levels vary substantially in somatic tissues [11] and the abundance and genomic distribution of 5hmC is dramatically altered during development [8, 12, 13]. Notably, 5hmC has been purported to play a key role as an intermediate in DNA demethylation; this may occur either passively during DNA replication [14], or actively through base excision repair of one or more oxidized intermediates [15, 16]. Other studies, however, regard 5hmC as a distinct epigenetic mark with a characteristic function independent of DNA demethylation [17–20]. The importance of the dynamic interplay between 5mC and 5hmC in maintaining normal DNA methylation patterns and gene expression and the causes and consequences of an imbalance are key questions yet to be answered.

Given the evidence that 5hmC plays a critical role in modulation of the DNA methylation landscape, it is essential to be able to distinguish 5hmC from 5mC and accurately detect and quantitate the levels of 5hmC at single-base resolution. In general, the levels of total 5hmC detected across the genome are approximately 14-fold lower than those of 5mC [21] although these levels vary substantially across tissue types: 5hmC is relatively abundant in brain tissues (~0.15–0.6% of total nucleotides) [22, 23], but is an order of magnitude lower (0.01–0.05%) in other mouse and human tissues [23–25]. In human cell lines, 5hmC abundance is at even lower levels (~0.007–0.009% of total nucleotides) [26]. Such low and variable abundance means that the method of detection has to be highly sensitive and specific for the 5hmC modification.

**Fig. 1:**
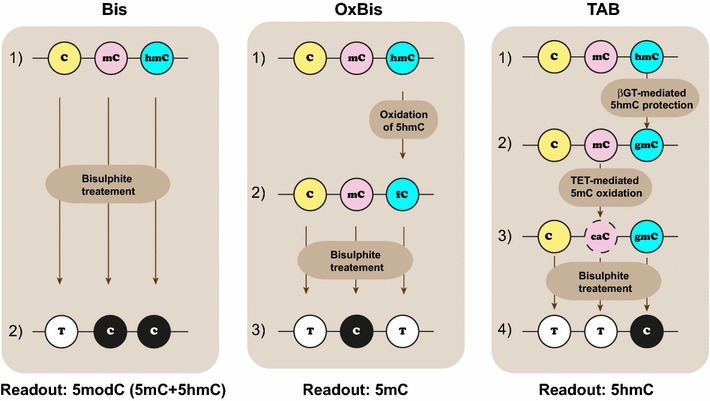
The workflow of conventional bisulphite (Bis), oxidative bisulphite (OxBis) and TET-assisted bisulphite (TAB) approaches for the detection of 5mC and 5hmC. Bisulphite treatment alone results in the conversion of unmethylated cytosine into uracil that will be read as thymine after PCR amplification, with both 5mC and 5hmC being read as cytosine. The readout of Bis is denoted as 5modC (5mC + 5hmC). The addition of an oxidation step prior to bisulphite treatment results in the conversion of 5hmC to 5fC that will be converted to uracil together with an unmethylated cytosine. Thus, the readout of OxBis is 5mC. In TAB, the first step involves β-glucosyltransferase-mediated protection of 5hmC with a glucose moiety, followed by TET-mediated oxidation of 5mC to 5caC, which will be converted to uracil. Thus, the readout of TAB is 5hmC

Importantly, bisulphite sequencing, the “gold standard” for 5mC analysis, does not distinguish 5mC from 5hmC, as both modified bases are resistant to conversion to uracil, in contrast to unmodified cytosine, which is converted to uracil (Fig. 1). This has precluded conventional bisulphite sequencing as a tool for 5hmC detection. Several approaches for hydroxymethylation mapping have been developed over the recent years. These include capture-based techniques, such as antibody-based hydroxymethylated DNA immunoprecipitation followed by sequencing (hMeDIP-seq) [6, 27, 28], and enrichment by hydroxymethyl selective chemical labelling (hMeSeal) [26]. These affinity-based methods have been widely used and provided the first genome-wide 5hmC profiles and biological insights of 5hmC [6, 26–28]. However, such approaches have relatively low resolution and cannot quantitatively determine 5hmC abundance in a single-base resolution manner.

Single nucleotide 5hmC mapping approaches have also been developed. The two best-described approaches are whole-genome oxidative bisulphite in combination with conventional bisulphite sequencing (WG Bis/OxBis-seq) [29] and TET-assisted bisulphite sequencing (TAB-seq) [30]. The principle of WG Bis/OxBis-seq relies on the specific oxidation of 5hmC by potassium perruthenate to form 5-formylcytosine (5fC) and/or 5-carboxylcytosine (5caC). 5fC and 5caC behave as unmethylated cytosines during bisulphite conversion. Therefore, the readout of OxBis-seq is specific for 5mC and does not contain the 5hmC fraction (Fig. 1a). Hence, subtraction of OxBis-seq readout (5mC) from the conventional Bis-seq readout (5mC + 5hmC) evaluates the hydroxymethylated proportion at a single CpG site (Bis-seq (5mC + 5hmC) − OxBis-seq (5mC) = 5hmC). For clarity, we use 5modC to denote Bis-seq readout (i.e. total methylation: 5modC = 5mC + 5hmC).

TAB-seq, on the other hand, gives a *direct* readout of 5hmC. The first step of TAB reaction includes protection of 5hmC residues with a glucose moiety implemented by β-glucosyltransferase (β-GT), whereas 5mC remains unprotected. Subsequent TET-mediated oxidation of 5mC, but not “protected” 5hmC, to 5fC/5caC followed by bisulphite treatment results in a conversion of 5fC/5caC and unmethylated cytosines to uracils, whereas hydroxymethylated cytosines remain as cytosines (Fig. 1b). Thus, while TAB-seq provides direct single nucleotide 5hmC profiling, OxBis-seq still requires conventional bisulphite (Bis-seq) to be performed in parallel to enable simultaneous 5hmC and 5mC single nucleotide readouts. These single nucleotide approaches also have been used for 5hmC interrogation on the HumanMethylation450 (HM450K) BeadChip arrays [31, 32]. Similar to TAB-seq, HM450K-TAB provides a direct readout of 5hmC, while HM450K Bis and OxBis arrays must be performed in parallel. Applying these single nucleotide approaches for whole-genome 5hmC mapping is ideal; however, the need for deep sequencing coverage for accurately resolving the 5hmC levels imposes restrictions and limitations on the widespread use of WG Bis/OxBis-seq and TAB-seq for mammalian genomes.

Here, we evaluate and compare three commonly used whole-genome 5hmC profiling approaches using WG Bis/OxBis-seq, HM450K arrays coupled with oxidative bisulphite (HM450K Bis/OxBis) and antibody-based immunoprecipitation of hydroxymethylated DNA (hMeDIP-seq). We also validate 5hmC at single nucleotide resolution using TAB-seq of selected candidate genomic regions. The comparisons were made on adult frontal lobe cerebellum DNA, as the highest levels of 5hmC have been reported in adult brain DNA [6, 33]. We highlight the advantages and caveats of 5hmC profiling methods and show that the interpretation of 5hmC data can be significantly influenced by the sensitivity of the method.

## Results

### Hydroxymethylation profiling by whole-genome Bis-seq/OxBis-seq

To compare the different technologies for analysis of whole-genome 5hmC profiling, we first performed 5hmC profiling using WG Bis/OxBis-seq, which allows interrogation of both 5hmC and 5mC at single nucleotide resolution. Human frontal lobe DNA, spiked with M.SssI CpG-methylated λDNA and fully hydroxymethylated 5hmC APC controls (“Methods” section), was treated with conventional bisulphite and oxidative bisulphite (CEGX TrueMethyl reagents), followed by library preparation and sequencing (Additional file 1: Table S1, Additional file 2: Figure S1; bisulphite conversion efficiency 98.30–99.76%, 5hmC oxidation efficiency 99.33%). The 5mC profile is a direct readout of Bis-seq, whereas the 5hmC profile is deduced by subtraction of the OxBis-seq readout from Bis-seq (Bis-OxBis).

**Fig. 2:**
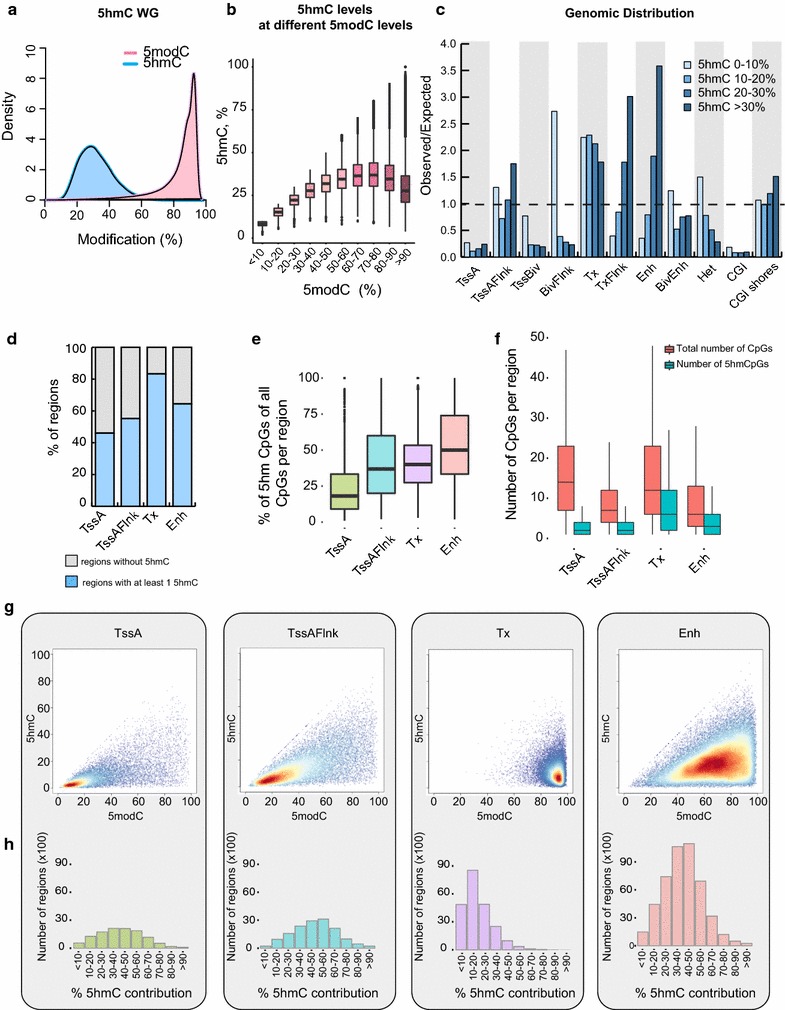
DNA methylation and hydroxymethylation profiling by whole-genome Bis-seq/OxBis-seq. **a** Methylation density plot showing the distribution of 5hmC and 5modC levels. Both 5modC and 5hmC density plots include CpG sites showing significant hydroxymethylation. **b** The relationship between the hydroxymethylated fraction and total methylation levels at each CpG site. CpG sites were binned into groups based on the total methylation levels, and the distribution of hydroxymethylation levels was calculated for each of these groups. **c** *Bar plot* showing observed over expected by chance enrichment of CpGs with different 5hmC levels at multiple genomic locations. Genomic regions comprise of Brain Frontal Lobe ChromHMM features as well as CpG islands and CpG island shores. **d** The percentage of genomic regions harbouring no hydroxymethylated CpG sites (*grey*) and those harbouring at least one hydroxymethylated CpG site (*blue*). **e** The proportion of hydroxymethylated CpGs of the total number of CpGs per genomic region. For each region, the total number of CpGs and number of hydroxymethylated CpGs were calculated (Additional file 2: Figure S1D). From that, for each region, the percentage of hydroxymethylated CpGs of all CpGs was calculated and distribution of those percentages was plotted. **f** The distribution of the total number of CpG sites and the number of hydroxymethylated CpG sites per genomic region. **g** The relationship between average total methylation (5modC) (*x*-axis) and average hydroxymethylation (5hmC) (*y*-axis) at different genomic regions. Each *dot* represents a single region. Hydroxymethylation levels of CpG sites that did not pass the statistical significance criteria were assigned to zero. **h** The hydroxymethylation contribution to the average total methylation at different genomic regions. For each genomic region, the percentage of hydroxymethylation contribution to the total methylation was calculated; the numbers of regions with the corresponding 5hmC contribution were plotted

Of the CpG sites considered significantly hydroxymethylated (*p* value ≤0.05: see “Methods” section), we found that the majority show 5hmC levels of ~30% and high total methylation levels (5modC) of 80–90% (Fig. 2a). To explore whether total methylation levels of CpG sites (5modC) are in proportion to 5hmC levels, we binned CpG sites based on their 5modC levels and plotted the distribution of 5hmC levels. The data reveal a linear relationship between total CpG methylation and 5hmC, with 5modC levels <50%, but a nonlinear relationship with 5modC levels >50% (Fig. 2b). This observation is consistent with a dynamic interplay between 5hmC and 5mC at sites that display lower levels (<50%) of total methylation or higher “plasticity” across the genome. In contrast, the more extensively methylated sites (>50%) show a more stable and less “plastic” methylation state, due to the decreasing 5hmC/5mC ratio.

Next, to investigate the genomic distribution of 5hmC, we annotated CpG sites, according to the 5hmC levels, to Brain Frontal Lobe ChromHMM features [34, 35], as well as CpG islands and CpG island shores. We show that active and bivalent promoters (TssA and TssBiv) and CpG islands (CGI) are depleted of hydroxymethylated CpGs (Fig. 2c), coinciding with their prevalent unmethylated state. However, regions flanking active and bivalent promoters are enriched predominantly for CpGs with low 5hmC levels (<10%) (Fig. 2c), while transcribed regions (Tx), flanking transcribed regions (TxFlnk) and enhancers (Enh) possess the highest levels of 5hmC enrichment (Fig. 2c).

Finally, we asked whether the hydroxymethylated cytosines make a significant contribution to the overall total methylation levels (5modC) at different genomic elements. To address this question, we focused on four genomic regions: active promoters, regions flanking active promoters, transcribed regions and enhancers (TssA, TssAFlnk, Tx, Enh, respectively). For each genomic region harbouring at least one 5hmC site (Fig. 2d), we calculated the percentage of hydroxymethylated CpGs of all CpGs and plotted the distribution (Fig. 2e). The analysis revealed ~50% of CpGs at enhancers are hydroxymethylated (3 CpGs out of 6 on average), whereas only ~15% of CpG sites at active promoters are hydroxymethylated (2 CpGs out of 13 on average) (Fig. 2e, f). To assess the contribution of 5hmC to the total methylation in each region, we plotted average 5hmC per region versus average total methylation (Fig. 2g) and calculated the number of regions with different 5hmC contributions (Fig. 2h). Despite the fact that only 15% of CpG sites at active promoters (TssA) are hydroxymethylated, 5hmC accounts for more than ~50% of the total methylation at the vast majority of active promoters (Fig. 2g). Similarly, over half of enhancers and regions flanking active promoters show that 5hmC contributes significantly to the total average methylation (Fig. 2h). However, most of the transcribed regions have low 5hmC levels relative to the total average methylation (Fig. 2h). Overall, these data reveal that 5hmC, when present, makes a substantial contribution to the total methylation levels at different genomic regions in brain DNA, highlighting the importance of discerning 5hmC from total methylation (5modC) in analysis of DNA methylomes from brain tissue.

### Methylation and hydroxymethylation profiling using Infinium HumanMethylation450 methylation arrays

**Fig. 3:**
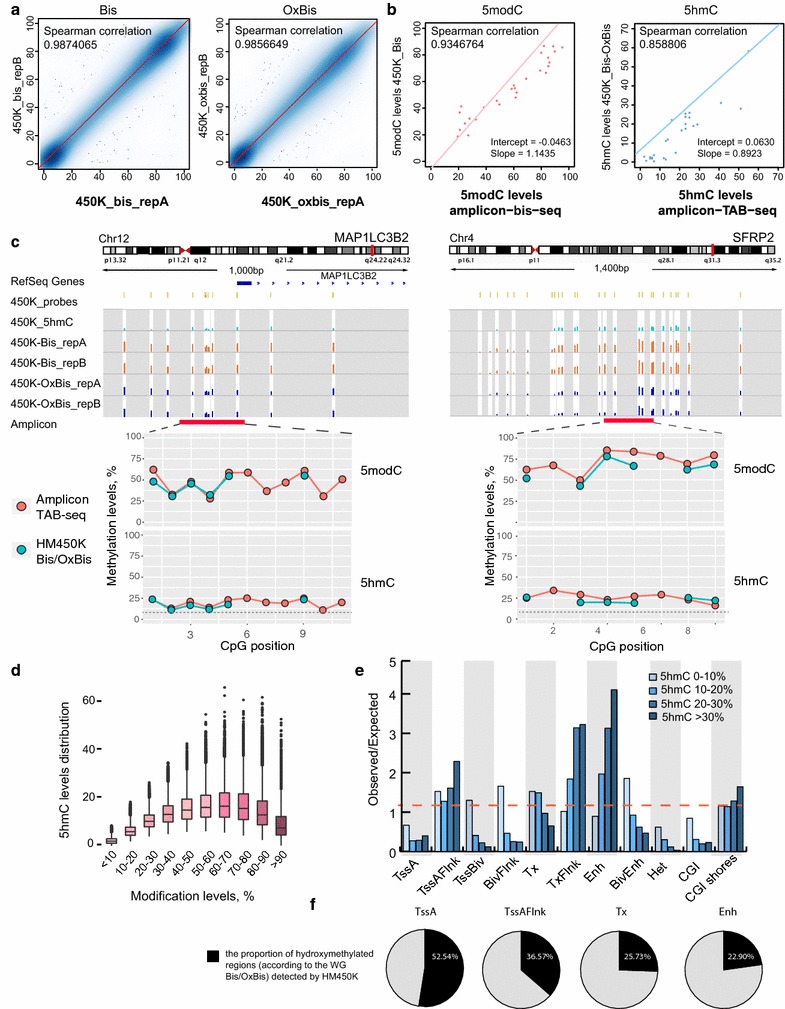
DNA methylation and hydroxymethylation profiling by HM450K Bis/OxBis. **a** *Scatter plots* showing the high correlation between HM450K Bis (*left*) and HM450K OxBis (*right*) replicates. Each *dot* (smoothed) reflects each probe 5modC (*left*) or 5mC (*right*) levels detected by two technical replicates (*x-* and *y*-axes). Spearman’s correlation 0.987406 (*left*) and 0.985664 (*right*), respectively. **b**, **c** 5hmC and 5modC MiSeq amplicon validation of candidate loci using Bis- and TAB-Seq. **b** *Scatter plots* showing the correlation of 5modC (*left*) and 5hmC (*right*) levels between HM450K and amplicon Bis- and TAB-seq. Each *dot* represents single CpG site/probe. The *pink* and *blue* regression *lines* show the intercept and slope of the plots. **c** HM450K screen shots of candidate regions, showing 450K_5hmC, 450K_Bis, 450K_OxBis. Amplicon validation of these genomic regions shows agreement in total methylation (5modC) and hydroxymethylation levels detected by HM450K Bis/OxBis and loci-specific Bis/TAB-seq, respectively. *Red dots* depict 5modC (*top*) and 5hmC (*bottom*) levels of each CpG site detected by loci-specific Bis-/TAB-seq, respectively. *Green dots* depict 5modC (*top*) and 5hmC (*bottom*) levels of HM450K CpG probes on the array. **d** The relationship between hydroxymethylation and different levels of total methylation detected by HM450K. CpG probes were binned into groups based on the total methylation levels, and the distribution of hydroxymethylation levels was calculated for each of these groups. **e** *Bar plot* showing observed over expected by chance enrichment of CpGs with different 5hmC levels (0–10, 10–20, 20–30, >30%) at multiple genomic locations; computationally derived chromatin segmentation (ChromHMM) of Brain Frontal Lobe genome, as well as CpG islands and CpG island shores. **f** *Pie charts* showing the proportion of genomic regions defined as hydroxymethylated by HM450K Bis/OxBis compared to the total number defined as hydroxymethylated according to the WG Bis/OxBis-seq. A region is considered hydroxymethylated if it contains >1 significantly hydroxymethylated CpG site

Infinium HumanMethylation450 BeadChip (HM450K) arrays have been widely used to study genome-wide 5mC DNA methylation profiles [36, 37], but more recently the technology has been adjusted to assess 5hmC [31, 32]. Here, we used the HM450K array in conjunction with OxBis to interrogate both 5mC and 5hmC. Frontal lobe adult DNA was treated with conventional bisulphite and oxidative bisulphite reaction, respectively, followed by hybridization to HM450K arrays (HM450K Bis and HM450K OxBis). Arrays were performed in duplicate with technical replicates showing high correlation (Spearman correlation of 0.987 and 0.986 for HM450K Bis and HM450K OxBis replicates, respectively (Fig. 3a). Of 482,422 cytosines interrogated on the array, 175,000 CpG sites possess significant hydroxymethylation levels (i.e. probes showing a significant difference between Bis and OxBis: adjusted *p* value ≤0.05: see “Methods” section).

We validated 5hmC levels detected by HM450K Bis/OxBis using amplicon TAB-seq of selected loci: 4 regions showing significant hydroxymethylation and 3 regions that showed no significant hydroxymethylation by HM450K Bis/OxBis (Fig. 3c, Additional file 3: Figure S2). TET-mediated 5mC oxidation efficiency of M.SssI CpG-methylated λDNA was 98.74% and *β*-glucosyltransferase-mediated protection of fully hydroxymethylated 5hmC pUC18 was ~100% (“Methods” section; Additional file 2: Figure S1). In parallel with TAB-seq, we performed conventional bisulphite sequencing to validate total methylation levels of those loci. Since a subset of CpG sites in the selected regions correspond to HM450K probes, we were able to directly compare 5modC (5mC + 5hmC) and 5hmC levels detected by HM450K Bis/OxBis with TAB-seq (Fig. 3c, Additional file 3: Figure S2A). Spearman’s correlation of 5modC and 5hmC levels across overlapping CpG sites from all loci sequenced shows 0.935 and 0.859, respectively (Fig. 3b), confirming the high accuracy of cytosine hydroxymethylation detection by HM450K Bis/OxBis Infinium arrays. Notably, however, amplicon TAB-seq was more sensitive and could detect 5hmC levels (~<10%) in regions that were not detected by HM450K (Additional file 3: Figure S2B), highlighting that that 5hmC levels need to be >10% to be detected by HM450K Bis/OxBis.

Notably, loci-specific Bis- and TAB-seq, with sequencing coverage ~50,000× and average of 13 CpG sites per loci, can also be used to determine single-molecule 5modC (5mC + 5hmC) and 5hmC levels, respectively (Additional file 4: Figure S3). Interestingly, loci with intermediate 5modC levels (50–75%, *x*-axis) predominantly consist of molecules with highly heterogeneous 5modC (A, purple and green dots) and 5hmC (B, green dots) single-molecule modification patterns, while loci with very low (<25%, *x*-axis) or very high (>75%, *x*-axis) levels of average 5modC methylation show more homogeneous patterns of 5modC (A, red and oranges dots, respectively) and 5hmC (B, red dots). This suggests that the vast majority of individual brain cells in the population with intermediate levels of 5modC methylation are not homogenous but display mosaic 5hmC/5mC modification patterns.

Since HM450K methylation arrays interrogate only 1.8% of CpG sites and predominantly represent CpG island promoters [38], we wanted to explore whether the array results in biased 5hmC calling of CpGs with different total methylation levels or at different genomic locations. To address this, we binned HM450K CpG probes based on their 5modC levels and plotted the distribution of 5hmC levels at those CpG sites. The data reveal a linear relationship between total CpG methylation and 5hmC, with 5modC levels <50%, but a nonlinear relationship with 5modC levels >50%, and notably CpGs with the highest 5modC levels (90–100%) are depleted in the 5hmC fraction (Fig. 3d), in agreement with WG Bis/OxBis-seq (Fig. 2b). The genomic distribution of 5hmC interrogated by HM450 also shows 5hmC observed/expected enrichment at regions flanking Tss (TssAFlnk), flanking transcribed regions (TxFlnk) and enhancers (Enh), and depletion from active promoters (TssA) and CpG islands (CGI) (Fig. 3e) in agreement with WG Bis/OxBis-seq (Fig. 2c). 5hmC was interrogated by HM450 at ~53% of TssA and 37% of TssAFlnk; however, a smaller proportion of hydroxymethylated Enh and Tx regions (23 and 26%, respectively) are detected by HM450K (Fig. 3f).

### Comparison of HM450K and whole-genome Bis-seq/OxBis-seq for 5hmC mapping

**Fig. 4:**
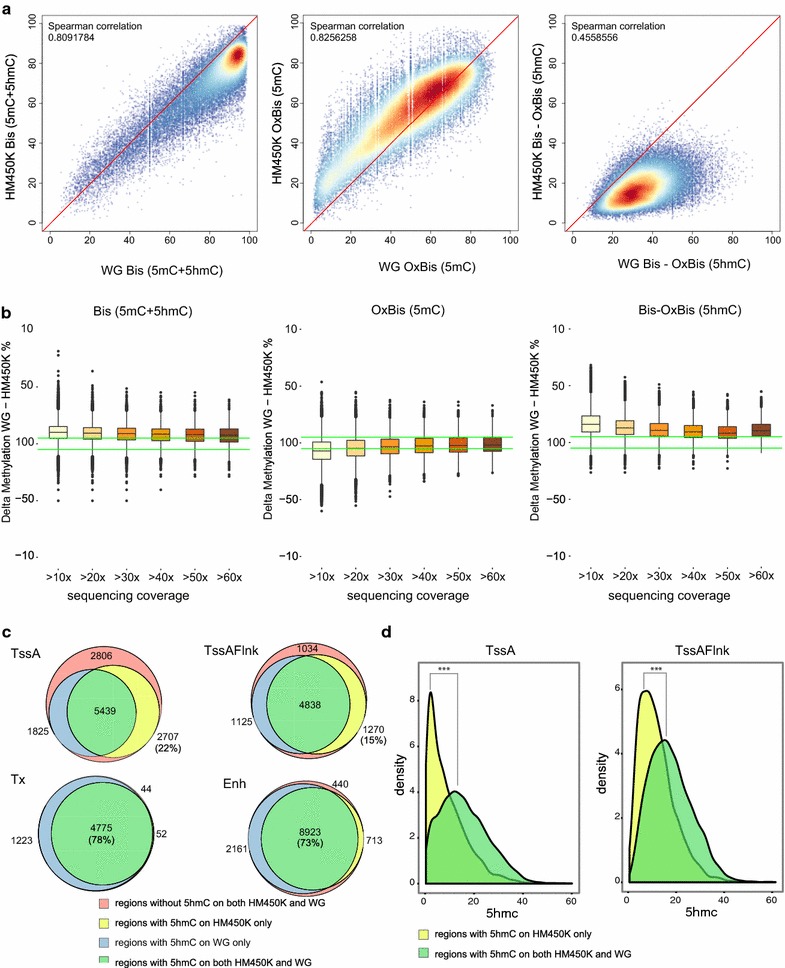
Comparative evaluation of HM450K Bis/OxBis and whole-genome Bis-/OxBis-seq for 5hmC profiling. **a** *Scatter plots* showing the correlation of 5modC (Bis, *left*), 5mC (OxBis, *middle*) and 5hmC (Bis-OxBis, *right*) between WG and HM450K Bis/OxBis across CpG sites (*n* = 42,537) considered as significantly hydroxymethylated by both approaches. Spearman’s correlation is indicated on each *scatter plot*. **b** The agreement in 5modC, 5mC and 5hmC levels detected by WG and HM450K Bis/OxBis as a function of WG sequencing coverage. The difference in methylation calling is plotted along the *y*-axis for each bin with defined sequencing coverage indicated (>10×, >20×, >30×–>60×). *Green lines* indicate ± 5% difference in methylation value detected between approaches (WG-HM450K). **c** *Venn diagrams* show the overlap of hydroxymethylated regions between WG and HM450K Bis/OxBis. Genomic regions with at least one CpG probe and at least 10× WG sequencing coverage were chosen for this analysis (11,625 of 25,235 total TssA; 14,945 of 27,078 total TssA_Flnk; 22,249 of 26,707 total Tx; 47,082 of 73,063 total Enh). Of those, the number of regions with at least one hydroxymethylated CpG according to HM450K only, WG only or both approaches was calculated and overlap was plotted. **d** The distribution of maximal hydroxymethylation values at the regions identified as hydroxymethylated according to the HM450K only (*yellow*) and according to the both WG and HM450K (*green*). For the active promoters (TssA), the median of the max(5hmC) distribution is 0.067 for the *yellow* group and 0.147 for the *green* group. For the regions flanking active promoters (TssA Flank), the median of the max(5hmC) distribution is 0.105 for the *yellow* group and 0.167 for the *green* group. The difference between the distributions of maximal 5hmC values between *yellow* and *green* groups of regions is statistically significant as determined by Kruskal–Wallis nonparametric test (*p* < 0.01)

Next, we compared the performance of 5modC and 5hmC profiling using HM450K Bis/OxBis arrays with WG Bis/OxBis-seq. Of all HM450K probes, 207,125 had sequencing coverage greater than 10× in both WG Bis-seq and OxBis-seq. Bis-seq and OxBis-seq values showed a high correlation between WG and HM450K data (Spearman’s correlation 0.907 and 0.914, respectively; Additional file 5: Figure S4). In addition, we determined the CpG sites common to both approaches with significant hydroxymethylation in both WG Bis/OxBis-seq and HM450K (42,537 probes) and compared the correlation of 5modC and 5hmC levels (Fig. 4a, Additional file 5: Figure S4). We found a good correlation between the platforms for 5modC (Bis) and 5mC (OxBis) methylation (Spearman’s correlation 0.809 and 0.826, respectively). However, 5modC displays a greater discrepancy at high methylation levels (>80%) with WG Bis-seq exceeding HM450K (Fig. 4a, Additional file 5: Figure S4). Such discordance between WG sequencing and HM450K at high 5modC levels has been reported previously, highlighting the need for improved normalization strategies for HM450K analysis [39].

We next addressed the effect of sequencing coverage and found that increasing sequencing depth (>10×–>60×) does not improve the concordance between WG Bis-seq and HM450K Bis (Fig. 4b, left). However, increasing sequencing depth improves agreement between the methods for 5mC (OxBis) methylation comparison (Fig. 4b, middle). In contrast, correlation of 5hmC levels between the two approaches does not improve with greater sequencing coverage (Fig. 4b, right) possibly due to the disagreement between WG and HM450K at high methylation levels.

We next determined whether the same genomic regions are defined as hydroxymethylated by both approaches. To this end, we took genomic regions covered by at least one HM450K probe, and at least 10× coverage on WG Bis/OxBis-seq and only considered hydroxymethylation of those CpGs for the subsequent analysis. Of these regions, we defined hydroxymethylated regions (>1× 5hmC CpG site) according to HM450K only, WG Bis/OxBis only or both. We found that the vast majority of enhancers (73%) and transcribed regions (78%) are defined as hydroxymethylated by both approaches (Fig. 4c, green). The presence of hydroxymethylated regions defined by WG only (Fig. 4c, blue) is potentially due to the 5hmC undercalling of HM450K. Such agreement is lower for the active promoters and flanking regions (Fig. 4c, green), where a substantial proportion of regions (22 and 15%, respectively) are not identified as hydroxymethylated on the WG (Fig. 4c, yellow). More detailed analysis revealed that regions identified as hydroxymethylated on HM450K only (yellow) possess lower levels of 5hmC compared to regions identified as hydroxymethylated by both approaches (Fig. 4d, green). This highlights that regions with lower 5hmC require higher sequencing coverage to detect 5hmC in the WG Bis/OxBis-seq data and therefore have not been detected as hydroxymethylated in the WG Bis/OxBis-seq data.

### Hydroxymethylation profiling by hMeDIP-seq

One of the advantages of Bis/OxBis-seq and TAB-seq for hydroxymethylation mapping is that these methods quantitatively resolve 5hmC at a single nucleotide resolution; however, the depth of sequencing needed to yield meaningful results is generally not cost-effective. An alternative approach for genome-wide 5hmC profiling is antibody-based enrichment of 5hmC, hMeDIP-seq, which is widely used due to its ease of use and cost-effectiveness. We performed hMeDIP on the same adult brain DNA (in duplicate), followed by library preparation and sequencing on HiSeq2500 to compare 5hmC calling with WG Bis/OxBis-seq and HM450K Bis/OxBis arrays.

**Fig. 5:**
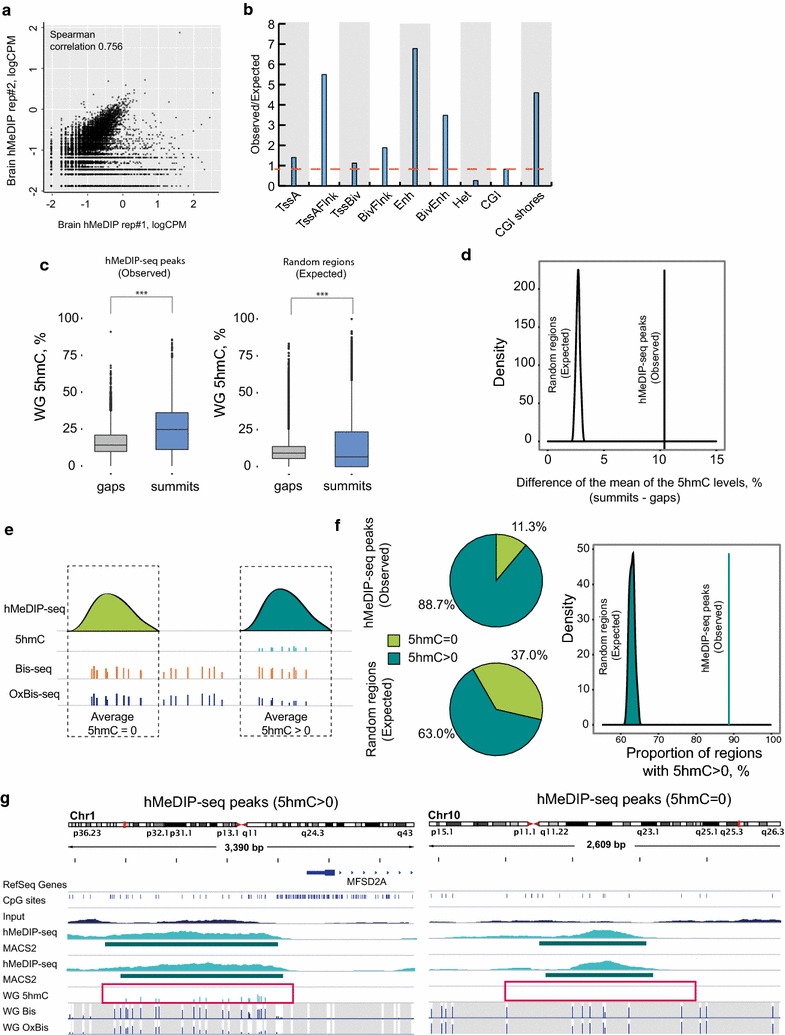
hMeDIP-seq hydroxymethylation profiling in the brain. **a** *Scatter plot* showing the correlation between hMeDIP-seq replicates. For 300-bp genomic tiles logCPM (count per million) values were calculated for each replicate. Each *dot* represents one genomic tile. **b** *Bar plot* showing observed over expected by chance enrichment of 5hmC peaks detected by hMeDIP-seq at multiple genomic locations; computationally derived chromatin segmentation (ChromHMM) of Brain Frontal Lobe genome, as well as CpG islands and CpG island shores. **c**, **d** Correlation of 5hmC profiling between WG Bis/OxBis and hMeDIP-seq. **c** The distribution of WG-derived 5hmC levels at hMeDIP summits expanded ± 150 bp and gaps between them (observed, *left*). Random permutation of hMeDIP summits and gaps and 5hmC levels distribution (expected, *right*). The differences between gaps and summits are statistically significant as determined by Kruskal–Wallis nonparametric test (*p* < 0.01). **d** The mean of the differences of 5hmC levels between hMeDIP-seq summits and gaps (observed) and the distribution of the mean for the permuted summits and gaps (expected). **e** Schematic representation of hMeDIP-seq peaks with no 5hmC detected by WG Bis/OxBis-seq (average 5hmC = 0) and with 5hmC detected by WG Bis/OxBis-seq (average 5hmC > 0). **f** *Pie charts* showing the proportion of real hMeDIP peaks (*top*) and randomly permuted hMeDIP peaks (*bottom*) with and without 5hmC detected by WG Bis/OxBis-seq. The distribution of the proportion of randomly permuted hMeDIP peaks with 5hmC (expected) and the actual proportion of hMeDIP peaks is shown on the *right*. For the analysis, hMeDIP-seq peaks with all associated CpGs having at least 10× WG coverage have been selected, which accounts for approximately 9000 peaks. **g** Genomic regions showing specific (*left*) and non-specific (*right*) hMeDIP-seq peaks

To first compare signal enrichment between replicates, we binned the genome into 300-bp tiles and calculated logCPM values per bin. The data reveal a high correlation between replicates at high logCPM values with overall Spearman’s correlation of 0.756 (Fig. 5a). MACS2 peak calling resulted in 49,292 hMeDIP-seq peaks with enrichment at regions flanking active TSSes, enhancers and CpG island shores (Fig. 5b), similar to the distribution of 5hmC detected by WG (Fig. 2c) and HM450K Bis/OxBis (Fig. 3e). To next assess the specificity of antibody-based 5hmC detection, we compared the data with WG Bis/OxBis-seq single nucleotide 5hmC mapping. We expanded each hMeDIP peak summit 150-bp up- and downstream (summits; Fig. 5c) and calculated average hydroxymethylation from WG Bis/OxBis-seq data and compared this to 5hmC at the regions not captured by hMeDIP-seq (gaps: Fig. 5c). The data revealed that hMeDIP peaks have significantly higher 5hmC levels (>10%) compared to the regions not captured by hMeDIP-seq (gaps; Fig. 5c, left). Random permutation of the hMeDIP peak summits and gaps does not result in the same trend (Fig. 5c, right). The mean of the differences of 5hmC levels between hMeDIP-seq peak summits and gaps (observed) is plotted alongside the distribution of the mean for the permuted summits and gaps (expected), showing the observed mean exceeding the expected mean (Fig. 5d), confirming the specificity of hMeDIP to 5hmC. Interestingly, splitting hMeDIP signal based on the relative enrichment over input signal read counts (logFC, “Methods” section) into low, medium and high categories did not show differences in 5hmC levels according to the WG Bis/OxBis (Additional file 6: Figure S5B). This highlights the semi-quantitative nature of hMeDIP-seq.

Finally, to interrogate potential non-specific binding of hMeDIP, we focused on hMeDIP peaks with all CpG sites having at least 10× sequencing coverage in WG Bis-seq and OxBis-seq (“Methods” section). We separated hMeDIP-seq peaks with detected average 5hmC (5hmC > 0, “Methods” section) and with no detected 5hmC (5hmC = 0) in WG Bis/OxBis-seq (Fig. 5e). The relative abundance of hMeDIP peaks with detected average 5hmC > 0 is 88.7%, revealing high specific hMeDIP binding (Fig. 5f, top). Random permutation of peaks resulted in a maximum of 65% of random peaks with 5hmC > 0, which is smaller than the observed value (88.7%) (Fig. 5f, right). We noted that 11.3% of 5hmC was not detected by WG Bis/OxBis (Fig. 5f), suggesting that either higher sequencing coverage of WG Bis/OxBis-seq is required and/or a degree of non-specific binding of hMeDIP (Fig. 5g, Additional file 6: Figure S5C). Together, the data show that hMeDIP-seq displays a high concordance with the WG Bis/OxBis-seq 5hmC profiling approach, even though ~10% hMeDIP peaks represent potentially non-specific enrichment.

### Profiling low abundance 5hmC in cell line DNA

**Fig. 6:**
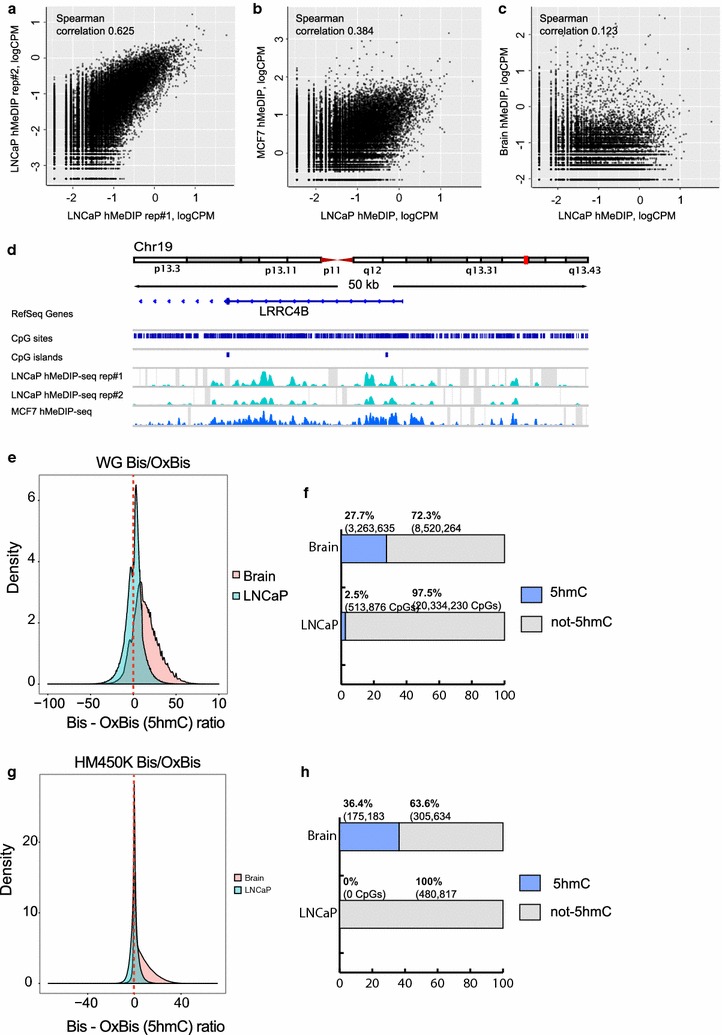
hMeDIP-seq hydroxymethylation profiling in cell line DNA. **a** *Scatter plot* showing the correlation between hMeDIP-seq replicates in LNCaP cells. For each genomic tile from one replicate the average enrichment score for the second replicate was calculated. Each *dot* represents one genomic tile. **b** *Scatter plot* showing the correlation between hMeDIP-seq in LNCaP cells and public hMeDIP-seq in MCF7 cells. **c** *Scatter plot* showing the correlation between hMeDIP-seq in LNCaP cells and brain hMeDIP-seq. **d** Genomic region showing the correspondence of hMeDIP-seq replicates in LNCaP cells as well as MCF7 cells. **e** Density plot showing the distribution of *p*_Bis_ − *p*_OxBis_ values in the Brain versus LNCaP WG Bis/OxBis data. **f** The percentages of significantly hydroxymethylated CpGs of all CpGs with at least 10× coverage on the WG Bis/OxBis in the Brain versus LNCaP. **g** Density plot showing the distribution of *p*_Bis_ − *p*_OxBis_ values in the Brain versus LNCaP HM450K Bis/OxBis data. **h** The percentages of significantly hydroxymethylated CpGs of all CpGs with at least 10× coverage on the HM450K Bis/OxBis in the Brain versus LNCaP

In contrast to adult brain DNA with a known high abundance of 5hmC, we next performed hMeDIP-seq on cancer cell line LNCaP DNA as cell lines are known to have low levels of 5hmC [18]. We observed less correlation (0.625) between replicates (Fig. 6a) than for the brain DNA (Fig. 5a). However, we show that the hMeDIP-seq peaks detected in prostate cancer cell line LNCaP show more correlation (0.384) with those identified in breast cancer cell line MCF7 (publically available: GSM1479831) (Fig. 6b, d) than brain DNA (0.123) (Fig. 6c), as expected between different DNA samples displaying low and high 5hmC content.

Next, we compared the performance of the single nucleotide 5hmC approaches (WG and HM450K Bis/OxBis) for brain and LNCaP DNA. Both single nucleotide 5hmC profiling approaches show the distribution of the 5hmC signal centred around zero with similar proportion of positive and negative values (Fig. 6e, g); however, the 5hmC signal in brain tissue is shifted towards positive values in both approaches (Fig. 6e, g). Since 5hmC is a subtraction of OxBis from the Bis signal, the distribution of 5hmC signal around zero reflects, per se, the error rates of both approaches. Further, the proportion of significantly hydroxymethylated CpGs of all CpGs (see “Methods” section) in the brain is 10 times greater than that of LNCaP cells (27.7 and 2.5% respectively), as detected by WG Bis/OxBis-seq (Fig. 6f). Importantly, HM450K Bis/OxBis did not detect any significant 5hmC in LNCaP cells (Fig. 6h), in contrast to the >35% of CpGs in the brain found to be hydroxymethylated by HM450K Bis/OxBis (Fig. 6h).

To rule out the possibility that the absence of 5hmC calling by the single nucleotide approaches is driven by the lack of sequencing coverage in WG Bis/OxBis and limitations of sensitivity of the HM450K array, we performed loci-specific TAB-seq with high (~50,000×) sequencing coverage. We chose regions that were detected by hMeDIP-seq (Additional file 7: Figure S6). While loci-specific TAB-seq showed 5hmC levels of <2–4% (Additional file 7: Figure S6), a proportion of the levels are comparable to the technique error rate (~1.8%, “Methods” section), suggesting potential non-specificity of hMeDIP-seq in genomes with low (<2–4%) 5hmC abundance.

These findings highlight that for DNA of high 5hmC abundance (5hmC levels >10%) hMeDIP-seq shows high specificity. However, in the absence or presence of very low levels of 5hmC (<~2–4%), such as in cell line DNA, hMeDIP-seq signals cannot be used as the representative of the actual 5hmC distribution without further validation.

## Discussion

For more than two and half decades, DNA methylation studies in higher organisms have relied on the use of bisulphite conversion technologies to study DNA methylation [38, 40]. However, it is now understood that these technologies do not distinguish between 5-methylcytosine (5mC) and 5-hydroxymethylcytosine (5hmC) [41, 42]. Since the discovery of 5-hydroxymethylcytosine and its potential role in modulating the DNA methylation landscape, there has been significant interest in defining the genome-wide distribution of 5hmC. Importantly, there is a critical need to distinguish 5hmC from 5mC and accurately detect and quantitate the levels of 5hmC at single-base resolution. A number of strategies have been developed to map 5hmC, including affinity-based approaches [6, 27, 28] and chemical modification approaches that allow single nucleotide resolution analyses [29, 30, 43]. In general, the abundance of total 5hmC detected across the genome is approximately tenfold to 100-fold lower [18] than that of 5mC, posing technical challenges for many approaches. Here, we have assessed three 5hmC genome-wide profiling approaches, using whole-genome bisulphite/oxidative bisulphite sequencing (WG Bis/OxBis-seq), Infinium HumanMethylation450 BeadChip arrays coupled with OxBis (HM450K Bis/OxBis) and antibody-based immunoprecipitation of hydroxymethylated DNA (hMeDIP-seq).

WG Bis/OxBis-seq approach enables analysis of the relationship between 5mC and 5hmC at the single nucleotide level. We were able to identify hydroxymethylated genomic regulatory regions (Fig. 2c, d), in particular at enhancer regions (Fig. 2e), highlighting the importance of taking 5hmC into consideration, especially when studying DNA methylation dynamics at distal regulatory regions in brain DNA. Interestingly, the vast majority of CpG sites with moderate modification levels possess the highest hydroxymethylation proportion compared to the more densely methylated CpGs that generally possess lower 5hmC signal (Fig. 2b). In addition, genomic regions with moderate methylation levels show mosaic 5hmC/5mC single-molecule methylation patterns. This observation is consistent with the hypothesis that 5hmC is prevalent at dynamic CpG sites showing higher “plasticity” across the genome [44]. Thus, the ability to discriminate 5mC and 5hmC on a genome-wide scale allows the contribution of 5hmC to total methylation to be elucidated, and to identify where regions are more dynamic or stably methylated.

Single nucleotide whole-genome 5hmC profiling is theoretically the most ideal approach to interrogating unbiased distribution of 5hmC across the genome. However, the use of WG approaches is restricted due to its high cost. To accurately assess 5hmC levels at single nucleotide resolution, 5hmC profiling requires higher sequencing depth, compared to 5mC methylation profiling since hydroxymethylation levels are more than a magnitude lower than DNA methylation levels [45]. As an alternative, HM450 BeadChip arrays have been modified to allow the detection of 5hmC separate from 5mC, used in conjunction with both oxidative [31] and TET-assisted bisulphite conversion [32]. Due to the fact that HM450K interrogates only 1.8% of all CpG sites in the human genome, 5hmC detection is restricted to the regions interrogated on the array. Using HM450K Bis/OxBis, we were able to detect hydroxymethylation at ~50% of promoters and ~20% of enhancers, which were identified as hydroxymethylated according to the WG Bis/OxBis-seq (Fig. 3f). However, since enhancers possess the highest enrichment of 5hmC among all genomic elements, a substantial proportion of potentially 5hmC-dependent functional genomic elements are not detected by HM450K. Importantly, the newly released Illumina EPIC array covers over 850,000 CpG sites, including >90% of the CpGs from the HM450 BeadChip and an additional 413,743 CpGs. The additional probes improve the coverage of regulatory elements, including enhancers, and therefore, this will offer significantly enhanced coverage of 5hmC-enriched genomic elements.

Overall, detection of hydroxymethylation at different genomic regions is highly concordant between approaches (Fig. 4c). However, HM450K Bis/OxBis possess a tendency of 5hmC signal underestimation, driven by CpG sites with extreme levels of total methylation, highlighting the necessity of improved normalization approaches of the array signal. This results in the “loss” of a significant number (~20%) of hydroxymethylated regions at different regulatory elements. On the other hand, a subset of promoters and flanking regions are identified as hydroxymethylated on HM450K Bis/OxBis *only* (Fig. 4d). Those regions possessed lower levels of 5hmC and therefore require higher sequencing coverage highlighting the higher sensitivity of the HM450 compared to the WG at a given sequencing coverage.

In contrast, antibody-based enrichment hMeDIP-seq of 5hmC has been widely used as a relatively easy and cost-effective approach. Overall, we observed a good agreement between hMeDIP-seq and WG Bis/OxBis-seq 5hmC signal as well as high concordance in the patterns of 5hmC genomic distribution. Moreover, we showed that, despite the presence of 5hmC signal in the hMeDIP-seq in cell line DNA, neither HM450K Bis/OxBis nor WG Bis/OxBis-seq and loci-specific TAB-seq were able to detect any significant 5hmC signal. This finding, together with the 5hmC profiling in adult brain DNA, suggests that in the presence of high levels of 5hmC, hMeDIP-seq is reliable, whereas in the absence or presence of very low levels of 5hmC, such as cell line DNA, hMeDIP-seq signal is potentially subject to misinterpretation of 5hmC distribution. An alternative affinity-based approach, hMeSeal, has been used for cost-effective whole-genome 5hmC profiling [24, 26]. While it has been shown to be a reliable and sensitive approach [18, 26] and successfully performed on low input DNA material [46], a detailed comparative analysis would be required to assess its performance compared to single nucleotide 5hmC profiling techniques on a whole-genome scale.

## Conclusions

In this study, we provide a detailed comparison of three genomic 5hmC profiling approaches. 5hmC profiling with WG Bis/OxBis-seq provides the most comprehensive quantitative overview of 5mC and 5hmC distribution across the whole genome in cells displaying higher levels of 5hmC, such as brain tissue. HM450K Bis/OxBis provides a user-friendly, high-throughput and affordable approach for cells that display higher levels of 5hmC, but will miss regions not present on the array and shows undercalling of 5hmC signal driven by CpGs with high methylation. Finally, hMeDIP-seq is a widely used and accepted approach due to its ease of use and cost-effectiveness. However, it is semi-quantitative, does not allow single nucleotide resolution and has the potential for non-specific enrichment in DNA displaying low 5hmC levels. Overall, we find a high correlation of hMeDIP with both WG Bis/OxBis and HM450K Bis/OxBis. Ultimately the method of choice for whole-genome profiling of 5hmC will depend on the abundance of 5hmC, number and quantity of DNA samples, cost consideration, bioinformatics expertise and the question being addressed.

## Methods

### DNA samples

Adult Brain Frontal Lobe genomic DNA from a single donor was obtained from Banksia Scientific Company (Bulimba, Australia, Cat No D1234035). Genomic DNA from the prostate cancer cell line LNCaP was extracted using QIAamp DNA Mini kit (Qiagen, USA). LNCaP prostate cells were cultured as described previously [47].

### Whole-genome bisulphite and oxidative bisulphite sequencing

Adult brain genomic DNA was sheared to an average size of 800 bp; 200 ng was used for the bisulphite (Bis) and oxidative bisulphite (OxBis) reactions. To assess the efficiency of potassium perruthenate-mediated oxidation of 5hmC and the behaviour of 5mC, genomic DNA was spiked with 5-hydroxymethylated 338-bp PCR product of APC genomic locus and M.SssI λDNA, respectively (described in “Spike-in controls for WG Bis/OxBis and TAB-seq” section). The bisulphite and oxidative bisulphite reactions were performed according to the manufacturer’s instructions (CEGX TrueMethyl^®^ WG user guide v2). Library preparation and indexing were also carried out as described (CEGX TrueMethyl^®^ WG). Library quality was assessed with the Agilent 2100 Bioanalyzer using the high-sensitivity DNA kit (Agilent, CA, USA). DNA was quantified using the KAPA Library Quantification kit by quantitative PCR (KAPA Biosystems). Paired-end 150-bp sequencing was performed for each library on the Illumina HiSeqX platform using the HiSeq X™ Ten Reagent Kit v2.

### Infinium HM450K bisulphite and oxidative bisulphite beadchip arrays

DNA was treated in separate aliquots with CEGX Bis and OxBis reagents according to the manufacturer’s specifications (CEGX TrueMethyl^®^_UGuide). 2 μg DNA was first sheared to 10 kb and then purified on BioRad^®^ P6 Micro-Bio spin column. Two aliquots were subjected to oxidation and two to mock oxidation prior to the Bis treatment (see CEGX protocol). Following the Bis reaction, the samples were re-quantified using the Qubit ssDNA assay. A minimum sample concentration of 20 ng/μl was required for the next step of the 450K process to ensure 7 μl of bisulphite-converted sample contains 140–160 ng of bisulphite-converted DNA. 7 μl of recovered TrueMethyl template was used in the HM450K protocol with 1 μl of 0.4 N NaOH (see Infinium Methylation assay). All subsequent steps were completed according to Illumina Infinium HM450K beadarray chip instructions.

### Hydroxymethylation profiling by hMeDIP-seq

DNA was sonicated with Covaris to produce fragments in size range of 300–500 bp. Prior to hMeDIP procedure, Illumina adaptors were ligated to (5 × 1 μg) of fragmented DNA as described in the TruSeq LT DNA Sample Preparation kit, Illumina. The hMeDIP assay was performed according to the manufacturer’s instructions (Active Motif, hMeDIP, Cat No 55010). Briefly, 3 × 1 μg of fragmented adapter-ligated DNA was spiked with 50 ng of either unmethylated, 5mC methylated or 5hmC hydroxymethylated 338-bp PCR product of APC genomic locus. The DNA was denatured for 10 min at 95 °C and immunoprecipitated overnight at 4 °C with 4 μl of 5hmC polyclonal antibody (Active Motif Cat No 55010). To allow selective enrichment of immune-captured DNA fragments, the mixture was incubated with 25 μl of Protein G magnetic beads for 2 h at 4 °C prior to washing all unbound DNA fragments. The bound hydroxymethylated DNA was eluted, treated with proteinase K and purified by Phenol/chloroform/isoamyl alcohol extraction and ethanol precipitation. The specificity of the hMeDIP assay was validated by qPCR of the unmethylated, methylated and hydroxymethylated spike-in APC controls and dot blots.

### Bisulphite and TET-assisted bisulphite treatment for amplicon sequencing

#### Bisulphite reaction

Bisulphite reaction was performed using EZ DNA Methylation-Gold Kit (Zymo Research, USA, Cat No D5005) according to the manufacturer’s instructions. M.SssI λDNA (5mC) was used as a spike-in control (described in “Spike-in controls for WG Bis/OxBis and TAB-seq” section) to assess the efficiency of bisulphite conversion reaction.

#### TET-assisted bisulphite treatment

TET-assisted bisulphite treatment was performed using the 5hmC TAB-seq Kit (WiseGene, USA, Cat No K001) according to the manufacturer’s instruction. Briefly, 1 μg genomic DNA was sonicated to the size of approximately 2kbp according to the manufacturer’s instructions. After sonication, DNA was spiked with 10 ng (1%) M.SssI λDNA (5mC) control and 10 ng (1%) 5hmC pUC18 control DNA. The β-GT-based reaction was performed at 37 °C for 1 h and the DNA purified using QIAquick PCR Purification kit (Qiagen, USA, Cat No 28106) according to the protocol and eluted in 27 μl water. The eluted DNA was split into two separate reactions to ensure no more than 300 ng DNA per TET1-based oxidation reaction. The TET1 oxidation reaction was performed at 37 °C for 1 h, followed by the treatment of 1 μl of proteinase K (20 mg/ml) at 50 °C for 1 h. The oxidized DNA was purified using QIAquick PCR purification kit (Qiagen, USA, Cat No 28106) and eluted in 50 μl water. TET1-oxidized DNA was then bisulphite-treated above using the EZ DNA Methylation-Gold kit (Zymo Research, USA Cat No D5005) as described in the protocol. Post-bisulphite conversion PCR amplification was performed in triplicate (4 ng/PCR); PCRs were pooled and purified using Wizard^®^ SV Gel and PCR Clean-Up System (Promega, USA, Cat No A9282). Library prep was performed following the instructions as per the Illumina TruSeq DNA sample prep kit (Cat No FC-121-2001) described below.

### Spike-in controls for WG Bis/OxBis and TAB-seq

Spike-in controls were used to assess the efficiency of the bisulphite, oxidative bisulphite and TET-assisted bisulphite reactions.

#### M.SssI λDNA (5mC)

We generated an in vitro CpG (5mC)-methylated λDNA control to (1) assess the bisulphite conversion efficiency of unmethylated cytosines (in a non-CpG context) to uracils and (2) to assess the efficiency of TET-mediated oxidation of 5mC in TAB-seq reaction. Unmethylated λDNA (Promega, USA, Cat No D1521) was sonicated to the average size of approximately 2 kbp according to the manufacturer’s instructions. 3 μg of sonicated DNA was used in methylation reaction using 4U of CpG methyltransferase M.SssI (New England Biolabs, USA, Cat No M0226S) in the presence of 640 μM SAM. Methylation reaction was allowed to proceed at 37 °C for 2 h and was stopped by heating at 65 °C for 20 min. CpG-methylated λDNA was purified using QIAquick PCR purification kit according to the protocol. After the completion of TAB reaction, 290-bp fragment of λDNA was amplified using following primers: forward 5′-TTTGGGTTATGTAAGTTGATTTTATG-3′ and reverse 5′-CACCCTACTTACTAAAATTTACACC-3′ (Additional file 8: Table S2). The PCR product was 3′-adenylated and ligated into the pGEM-T-easy plasmid (Promega, USA, Cat No A1360), followed by MiSeq amplicon sequencing.

For WG Bis/OxBis, the efficiency of bisulphite conversion was 98.30% for Bis and 99.76% for OxBis. For TAB-seq, the efficiency of bisulphite conversion was 99.58% and TET-mediated 5mC-to-T oxidation efficiency was 98.74% (Additional file 2: Figure S1C, D). The efficiency of M.SssI λDNA CpG methylation was assessed by clonal Sanger sequencing after the completion of the conventional bisulphite reaction, which was performed alongside.

## 5hmC pUC18 control

To assess the efficiency of β-GT-mediated protection of 5hmC, an in vitro hydroxymethylated pUC18 control was generated. 1.64-kbp region of pUC18 plasmid was amplified in the presence of 5-hydroxymethyl-dCTP, 5hmdCTP (Zymo Research, USA, Cat No D1045) using following primers: forward 5′-GCAGATTGTACTGAGAGTGC-3′ and reverse 5′-TGCTGATAAATCTGGAGCCG-3′ (Additional file 8: Table S2). After the completion of TAB reaction, 190-bp fragment of 1.64-kbp 5hmC pUC18 control was amplified using the following primers: forward 5'-GTAGATTGTATTGAGAGTGT-3' and reverse 5'-TACCCAACTTAATCGCCTTG-3' (Additional file 8: Table S2), followed by the clonal Sanger sequencing as described for the M.SssI λDNA control. Due to the contamination of 5hmdCTP with unmodified 5dCTP, the actual degree of hydroxymethylation of 5hmC pUC18 control had to be assessed by clonal Sanger sequencing after the completion of the conventional bisulphite reaction, which was performed alongside. The β-GT-mediated protection was ~100% as estimated using 5hmC-to-T conversion efficiency of 5hmC pUC18 control in TAB reaction normalized to that in the conventional bisulphite reaction (Additional file 2: Figure S1D).

## 5hmC APC control

To assess the efficiency of potassium perruthenate (KRuO_4_)-mediated oxidation of 5mC in the OxBis reaction, Illumina TruSeq DNA adapters were ligated to the commercially available 5hmC APC PCR product of APC genomic locus (Active Motif, USA, Cat No 55008). Briefly, 1 μg of 5hmC APC control was used for the end repair, A-tailing and ligation of Illumina adapter according to the Illumina instructions. After the completion of the OxBis reaction, 5hmC APC PCR product was amplified using PCR primer cocktail supplied by Illumina followed by the clonal Sanger sequencing as described for the M.SssI λDNA control (Additional file 2: Figure S1B). The KRuO_4_-mediated 5mC-to-5caC/(T) oxidation efficiency was 99.33% as estimated using 5hmC-to-5caC/(T) conversion efficiency of 5hmC pUC18 control in OxBis reaction normalized to that in the conventional bisulphite reaction.

### MiSeq amplicon TAB sequencing

Validation of candidate regions was performed on bisulphite- and TET-assisted bisulphite-treated DNA described above. First, PCRs were performed on a temperature gradient between 50 and 62 °C to achieve optimal amplification temperature. Each PCR was checked on an agarose gel for specific PCR products. To test for the amplification bias, we used the following bisulphite-treated control DNA: (1) human genomic blood DNA (Roche Cat No 11691112001) (unmethylated control DNA); (2) serological DNA from Chemicon (100% methylated control); (3) 50:50 mix of Roche and serological DNA under three different concentrations of MgCl_2_. For each PCR, optimal temperature and MgCl_2_ concentrations were determined as described previously [48].

PCRs of Adult Brain and LNCaP DNA (bisulphite or TET-assisted bisulphite treated) were performed in triplicate using optimized conditions (Additional file 8: Table S2). PCRs were pooled and purified using Wizard SV and PCR Clean-Up System (Promega, USA, Cat No A9282) and quantitated by Qubit. Library prep was performed following the instructions as per the Illumina TruSeq DNA sample prep kit (Cat No FC-121-2001). Briefly, 1000 ng of pooled amplicon input DNA was used for each library preparation. End repair, A-tailing and ligation of Illumina adapter to pooled PCR library were performed according to the Illumina instructions. After PCR clean-up, the library was quantified by Qubit, diluted to 10 nM according to Qubit, and accurately quantitated by KAPA SYBR FAST Universal qPCR (KAPA Biosystems, USA, Cat No KK4835) before being sequenced on the Illumina MiSeq™ sequencer (Illumina, CA, USA).

### Data analysis

Data processing and alignment was performed using in-house computational pipelines. Statistical analyses were conducted in the R statistical software.

#### Whole-genome Bis/OxBis sequencing data

Bisulphite reads were aligned to the human genome using version 1.2 of an internally developed pipeline, publicly available for download from http://github.com/astatham/Bisulphite_tools. Briefly, adaptor sequences and poor-quality bases were removed using TrimGalore (version 0.2.8, http://www.bioinformatics.babraham.ac.uk/projects/trim_galore/) in paired-end mode with default parameters. Bismark v0.8.326 was then used to align reads to hg19 using the parameters “-*p* 4 –bowtie2 –X 1000 –unmapped –ambiguous –gzip –bam”. PCR duplicates were removed using Picard v1.91 (http://broadinstitute.github.io/picard). Count tables of the number of methylated and unmethylated bases sequenced at each CpG site in the genome were constructed using bismark_methylation_extractor with the parameters “-*p* –no_overlap –ignore_r2 4 –comprehensive –merge_non-CpG –bedgraph –counts –report –gzip –buffer_size 20G”. The adult brain Bis and OxBis libraries had a total of 517,530,911 and 489,841,771 reads, respectively. Both libraries passed basic quality control checks with 89/90% alignment rate and 18×/16× mean coverage for adult brain Bis/OxBis, respectively.

To assess the level of 5hmC at a CpG locus, we compared the number of retained cytosines in Bis experiment to that in OxBis experiment. More specifically, we assumed that the number of retained cytosines follows a binomial distribution *NC* ~ Binomial (*N*, *p*), where *N* is the coverage and *p* is the proportion of modified cytosines. For the Bis experiment *p*_Bis_ = *p*_5mC_ + *p*_5hmC_ is the proportion of both 5mC and 5hmC modifications, whereas for OxBis experiment *p*_OxBis_ = *p*_5mC_ is the proportion of 5mC modifications. Thus, to assess the proportion of 5hmC modifications we used *proportion test*, as implemented in R’s prop.test() function, to estimate the difference between *p*_Bis_ and *p*_OxBis_, given *NC*_Bis_, *N*_Bis_, *NC*_OxBis_ and *N*_OxBis_.

Average coverage for Bis-seq and OxBis-seq was 18× and 16×, respectively (Additional file 1: Table S1). Before applying proportion test, we filtered out CpG loci having less than 10x coverage in either Bis or OxBis dataset. This reduced the number of tested CpG loci from 28,269,977 to 11,783,899 for adult brain data. CpGs with statistically significant difference between Bis and OxBis (*p* value <0.05, *p*_Bis_ − *p*_OxBis_ > 0) were considered as significantly hydroxymethylated resulting in 3,263,635 CpG sites. Only these CpG sites are considered in the subsequent analyses. For the calculations of the *average hydroxymethylation* per region, we discarded CpGs with *p* value ≥0.05 and *p*_Bis_ − *p*_OxBis_ > 10% as well as CpGs with *p*_Bis_ − *p*_OxBis_ < 0 and imputed CpGs with *p* value ≥0.05 and 0 < *p*_Bis_ − *p*_OxBis_ < 10% to zero.

We performed power calculations using R’s power.prop.test() function to determine power as a function of coverage in both Bis and OxBis experiments.

#### HM450K Bis/OxBis data

Two replicates of adult brain samples per treatment condition, Bis or OxBis, were profiled on Illumina’s HumanMethylation450K array [37]. The raw data were preprocessed and background normalized with Biconductor minfi package [49] using preprocessIllumina(…, bg.correct = TRUE, normalize = “controls”, reference = 1) normalization function. We used the limma Bioconductor package [49, 50] to perform differential methylation analysis between Bis and OxBis treatments to determine levels of 5hmC. We only considered probes for which there was a reliable methylation readout (detection *p* value <0.01) in all four samples. We then transformed *β*-values into *M*-values using logit transformation: $M = \log \left( {\frac{\beta }{1 - \beta }} \right)$. (To avoid extreme *M*-values, the *β*-values were capped at 0.01 and 0.99.) Standard limma workflow with unpaired contrast was then applied to computed *M*-values to call differentially methylated probes between Bis and OxBis and thus to determine levels of 5hmC. This analysis resulted in 175,183 probes having significant hydroxymethylation (*p* value <0.05, *p*_Bis_ − *p*_OxBis_ > 0).

#### TAB-seq data

Paired-end fastq files were obtained for each library and aligned to hg19 using bwa-meth (http://github.com/brentp/bwa-meth, arXiv:1401.1129). Downstream analysis was performed using the “ampliconAnalysis” function of the R package aaRon (http://github.com/astatham/aaRon). Data quality was checked by assessing the number of reads obtained and the bisulphite conversion efficiency per amplicon and per sample. All samples had high bisulphite conversion efficiency >98% and amplicons had >10,000× coverage. Percent hydroxymethylation/methylation at each CpG site was calculated.

#### hMeDIP sequencing data

Sequenced reads from hMeDIP immunoprecipitated and input control human brain samples were mapped to the reference human genome (hg19) with bowtie v.1.1.0 [51], allowing up to three mismatches. Reads mapping to multiple locations and/or deemed as PCR duplicates were filtered out. Reads were extended 300 bp and overlapped with the 300-bp tiling of the human genome to create a table of counts to be used for statistical modelling. Bins with low total number of reads (less than 20) across the three samples were removed from the analysis. Standard analysis flow as implemented in the edgeR Bioconductor package [52] was then applied to contrast read counts in hMeDIP samples to the input control. The library normalization step as implemented in calcNormFactors() was omitted and dispersion was set to 0.01. The same analysis was repeated for regions of 300 bp centred on summits of broad peaks identified with MACS2 algorithm [53]. Summit regions from two hMeDIP replicates were merged resulting in 137,598 regions used for the analysis. Of those, 137,373 regions had *p* value <0.05 and logFC > 0 (with the smallest logFC 1.391). Regions with FDR < 0.1 and logFC > 0 (smallest logFC 1.391) were called as having 5hmC modification resulting in 21,553 marked regions for summit-centred analysis. For the specificity analysis, we selected only hMeDIP-seq regions with all CpGs per region having at least 10× coverage in the WG Bis/OxBis resulting in 7563 regions. Next, we calculated average hydroxymethylation (as described in “Whole-genome Bis/OxBis sequencing data” section) per each region and separated into two categories: average 5hmC > 0 and 5hmC = 0. For LNCaP, hMeDIP replicates were merged resulting in 240,216 regions used for the analysis.

### Genome annotation

#### Genomic locations

Genomic coordinates (hg19) of CpG islands were obtained from UCSC genome browser. Genomic coordinates of CpG island shores were derived by taking ±2 -kb flanking regions around CpG islands.

#### ChromHMM annotations

A bed-formatted annotation file of chromatin states was downloaded from the Encode Roadmap (http://egg2.wustl.edu/roadmap/data/byFileType/chromhmmSegmentations/ChmmModels/coreMarks/jointModel/final/E073_15_coreMarks_dense.bb) [34, 35]. We used hyper-geometric testing to determine statistical significance of overlap between regional hypomethylated probes and the above functional annotations of the genome.

## Additional files

**Additional file 1. Table S1.** Summary of sequencing metrics.

**Additional file 2. Figure S1.** Spike-in controls showing the efficiency of the WG OxBis and loci-specific TAB reactions. **A**, **B** Each lollipop represents single CpG in M.SssI λDNA 5mC control and single cytosine in 5hmC pUC18 control. **C**, **D** The efficiency of TET-mediated oxidation of 5mC (**C**) and β-glucosyltransferase-mediated protection of 5hmC (**D**) of spike-in controls in loci-specific Bis/TAB-seq. **C** In vitro M.SssI CpG-methylated λDNA was used as a spike-in control to estimate the TET-mediated 5mC oxidation efficiency. Deep Bis-seq shows the degree of CpG methylation of M.SssI λDNA and TAB-seq shows the efficient oxidation of methylated CpGs, and therefore, very low methylation signal (TET-mediated 5mC-to-T oxidation efficiency) was calculated as the ratio of mC signal in TAB and Bis and was 98.74%; non-conversion rate of unmodified cytosine was 0.42%. **D** Region from pUC18 plasmid amplified in the presence of 5hm-dCTPs was used as a spike-in control to estimate the efficiency of β-glucosyltransferase-mediated 5hmC protection from the oxidation by TET enzymes. Deep Bis-seq shows the degree of cytosines hydroxymethylation of 5hmC pUC18 and deep TAB-seq shows the efficiency of protection (β-glucosyltransferase-mediated protection efficiency equals to 100%).

**Additional file 3. Figure S2.** Loci-specific Bis/TAB-seq for HM450K validation. Genomic regions showing agreement in total methylation and hydroxymethylation levels detected by HM450K Bis/OxBis and loci-specific Bis/TAB-seq, respectively. *Red dots* depict 5modC (*top*) and 5hmC (*bottom*) levels of each CpG site detected by loci-specific Bis/TAB-seq, respectively. *Blue dots* depict 5modC (*top*) and 5hmC (*bottom*) levels of HM450K CpG probes. **A** Regions with significant hydroxymethylation according to HM450K (SLC12A6_2: chr15: 34,628,635–34,628,921; PEG10: chr7: 94,285,834 94,286,118). **B** Regions with no hydroxymethylation according to HM450K. The selected negative regions had a similar range of total methylation values to positive regions and serve as a control to eliminate the differences in 5hmC detection that could be caused by different levels of total methylation (e.g. efficiency of TET-mediated oxidation).

**Additional file 4. Figure S3.** Single-molecule total methylation (**A**) and hydroxymethylation (**B**) patterns of each region (represented as one *vertical blue line*) were determined based on the deep loci-specific Bis/TAB-seq, respectively. Methylation patterns were separated into five groups based on the percentage of CpGs per region being methylated and/or hydroxymethylated (**A**) and hydroxymethylated (**B**). For each region (*vertical blue line*), the average total methylation (**A**) or hydroxymethylation (**B**) was calculated (*x*-axis) and the frequency of each pattern was plotted along the y-axis (summing up to 100%). Five different patterns were defined based on the proportion of methylated or hydroxymethylated CpGs of all CpGs per region (0, 0–10, 10–50, 50–80, 80–100%).

**Additional file 5. Figure S4.** WG Bis/OxBis-seq and HM450K Bis/OxBis correlation analysis. **A** Comparison of 5modC (Bis, *left*) and 5mC (OxBis, *right*) between WG and HM450K Bis/OxBis across CpG sites interrogated by the HM450K and having at least 10× coverage on the WG Bis/OxBis. **B** Boxplots showing the difference in 5modC (Bis, *left*), 5mC (OxBis, *middle*) and 5hmC (Bis-OxBis, *right*) between WG and HM450K Bis/OxBis at different levels of the corresponding modification. Only CpG sites (*n* = 42,537) considered as significantly hydroxymethylated by both approaches are included. **C** *Boxplots* showing the relationship between the total methylation levels according to the WG Bis/OxBis (*x*-axis) and the difference in 5mC (*left*) and 5hmC (*right*) between approaches. The difference is calculated as HM450K methylation value subtracted from the WG methylation value (*y*-axis).

**Additional file 6. Figure S5.** Validation of the hMeDIP-seq approach with the WG Bis/OxBis-seq. **A** The histogram showing the distribution of the hMeDIP-seq signal normalized to the input (logFC). The *colours* indicate the cut-offs set to depict low (log FC < 1.8), medium (log FC > 1.8 and <2.4) and high (log FC > 2.4) hMeDIP signal enrichment. **B** The distribution of WG-derived 5hmC levels at hMeDIP summits expanded ±150 bp binned based on their logFC values; and gaps between them. **C** *Screen shots* of genomic regions showing specific (above) and non-specific (below) hMeDIP-seq peaks.

**Additional file 7. Figure S6.** TAB-Seq amplicon validation of hMeDIP-seq in cell line DNA. *Screen shots* showing hMeDIP-seq peaks. WG Bis/OxBis data are shown. *Grey* denotes regions not covered >10× by WG Bis/OxBis. Locus-specific TAB-Seq validation showing mod C (5mC + 5hmC) and 5hmC across the amplicons with %5mC and %5hmC for each CpG site indicated.

**Additional file 8. Table S2.** Primers.

## Abbreviations

5hmC: 5-hydroxymethylcytosine
5mC: 5-methylcytosine
5modC: total methylation (5mC + 5hmC)
TAB-seq: TET-assisted bisulphite sequencing
Bis-seq: bisulphite sequencing
OxBis: oxidative bisulphite
WG: whole genome
