# Supplementary figures and images for: Comprehensive evaluation of genome-wide 5-hydroxymethylcytosine profiling approaches in human DNA
Source: Epigenetics Chromatin. 2017 Apr 20;10:16. doi: 10.1186/s13072-017-0123-7 (PMC5397694; doi:10.1186/s13072-017-0123-7)

**A**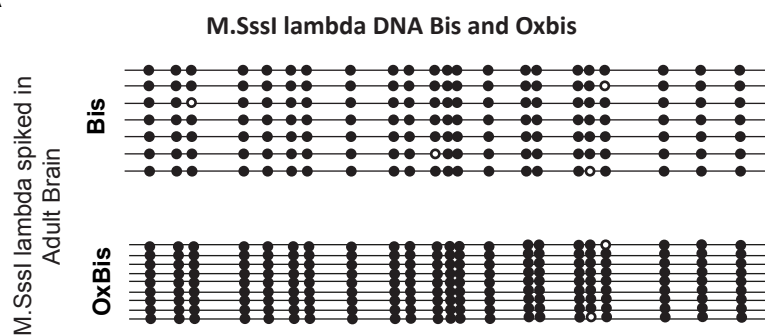**B**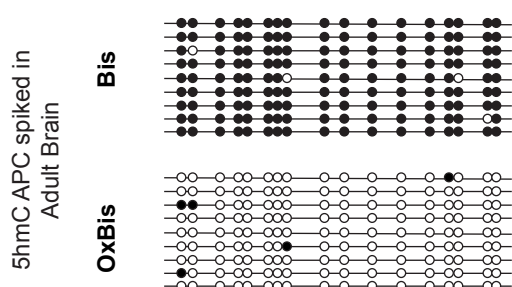**C**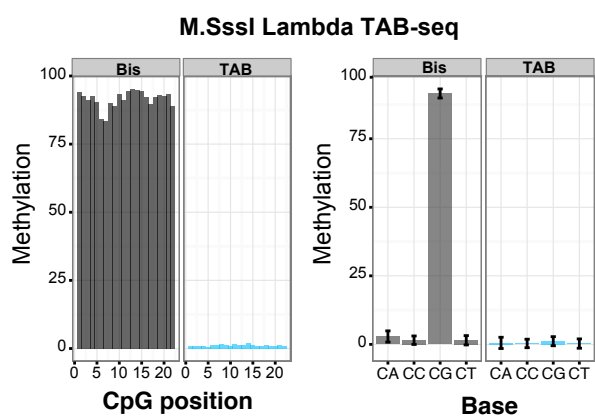**D**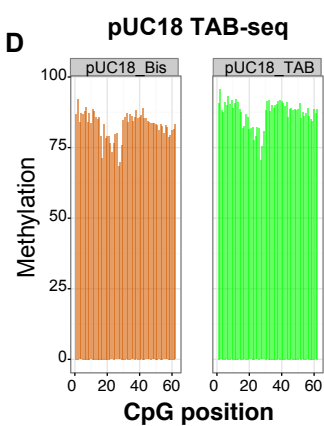

Supplement: Supplementary file 2 — Additional file 2. Figure S1. Spike-in controls showing the efficiency of the WG OxBis and loci-specific TAB reactions. A, B Each lollipop represents single CpG in M.SssI λDNA 5mC control and single cytosine in 5hmC pUC18 control. C, D The efficiency of TET-mediated oxidation of 5mC (C) and β-glucosyltransferase-mediated protection of 5hmC (D) of spike-in controls in loci-specific Bis/TAB-seq. C In vitro M.SssI CpG-methylated λDNA was used as a spike-in control to estimate the TET-mediated 5mC oxidation efficiency. Deep Bis-seq shows the degree of CpG methylation of M.SssI λDNA and TAB-seq shows the efficient oxidation of methylated CpGs, and therefore, very low methylation signal (TET-mediated 5mC-to-T oxidation efficiency) was calculated as the ratio of mC signal in TAB and Bis and was 98.74%; non-conversion rate of unmodified cytosine was 0.42%. D Region from pUC18 plasmid amplified in the presence of 5hm-dCTPs was used as a spike-in control to estimate the efficiency of β-glucosyltransferase-mediated 5hmC protection from the oxidation by TET enzymes. Deep Bis-seq shows the degree of cytosines hydroxymethylation of 5hmC pUC18 and deep TAB-seq shows the efficiency of protection (β-glucosyltransferase-mediated protection efficiency equals to 100%). [file 13072_2017_123_MOESM2_ESM.pdf]

**A**

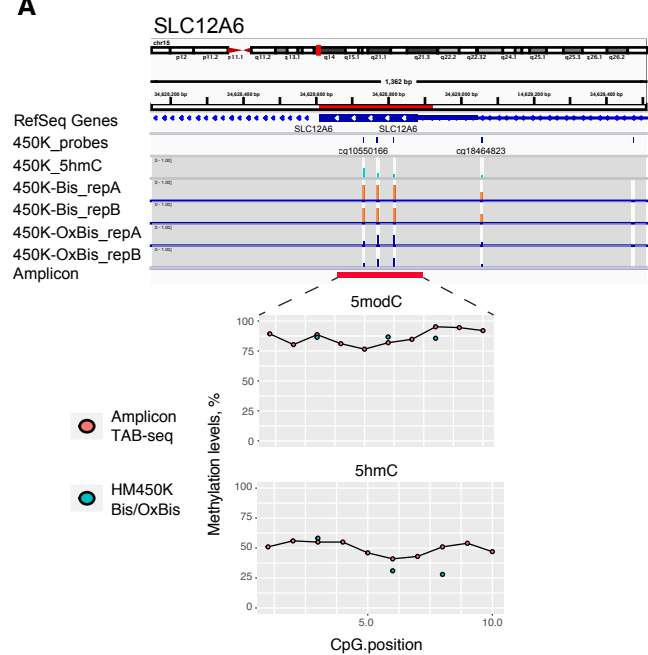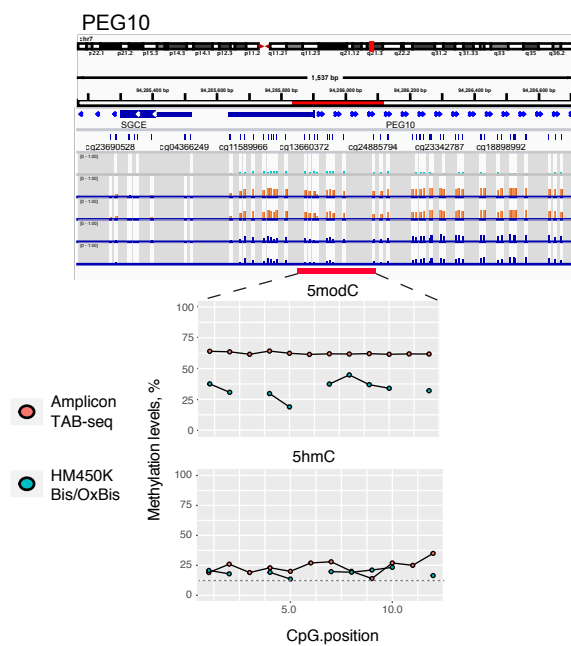

B

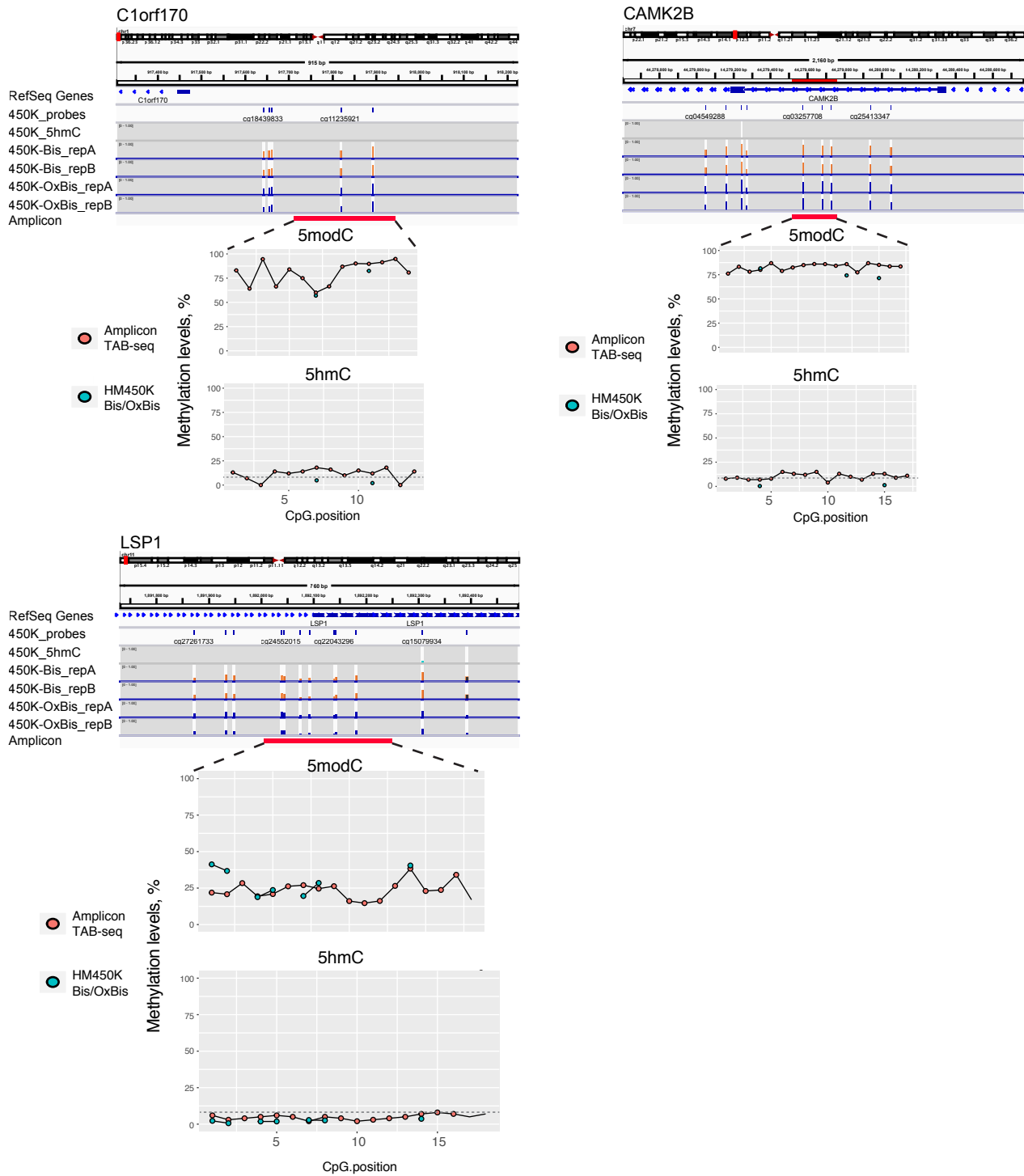

Supplement: Supplementary file 3 — Additional file 3. Figure S2. Loci-specific Bis/TAB-seq for HM450K validation. Genomic regions showing agreement in total methylation and hydroxymethylation levels detected by HM450K Bis/OxBis and loci-specific Bis/TAB-seq, respectively. Red dots depict 5modC (top) and 5hmC (bottom) levels of each CpG site detected by loci-specific Bis/TAB-seq, respectively. Blue dots depict 5modC (top) and 5hmC (bottom) levels of HM450K CpG probes. A Regions with significant hydroxymethylation according to HM450K (SLC12A6_2: chr15: 34,628,635–34,628,921; PEG10: chr7: 94,285,834 94,286,118). B Regions with no hydroxymethylation according to HM450K. The selected negative regions had a similar range of total methylation values to positive regions and serve as a control to eliminate the differences in 5hmC detection that could be caused by different levels of total methylation (e.g. efficiency of TET-mediated oxidation). [file 13072_2017_123_MOESM3_ESM.pdf]

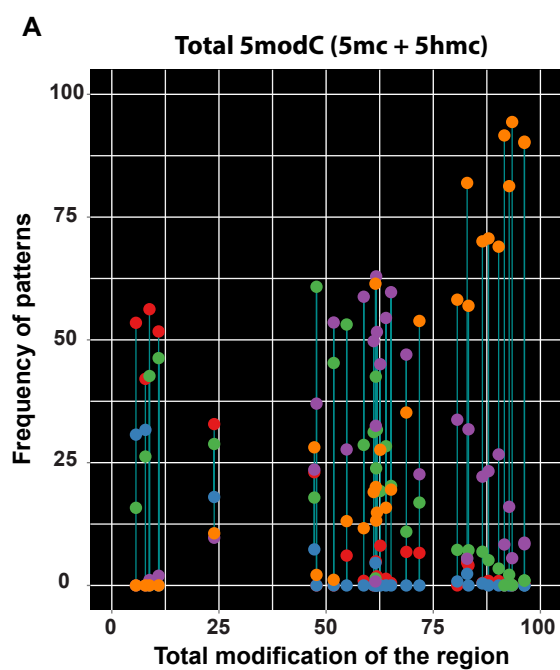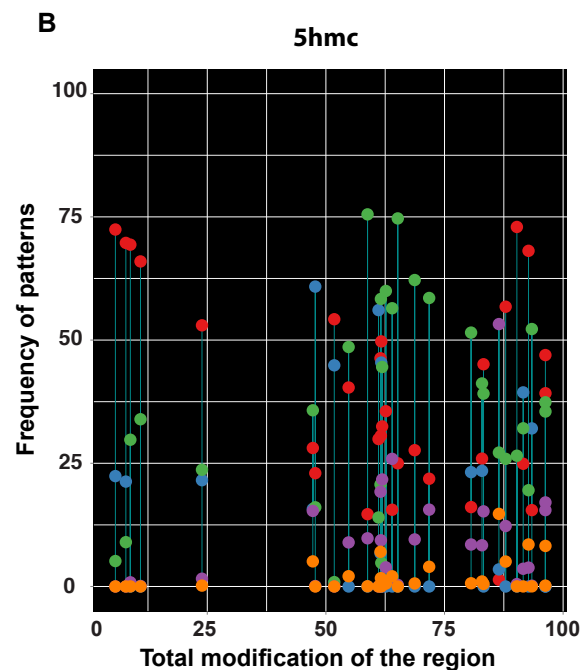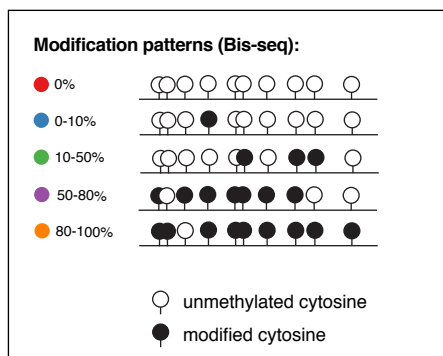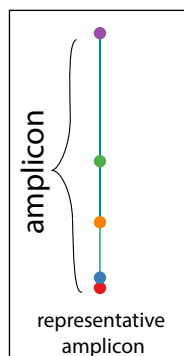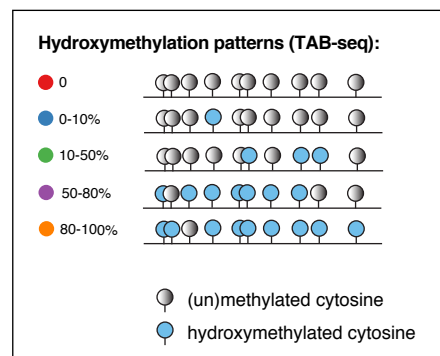

Supplement: Supplementary file 4 — Additional file 4. Figure S3. Single-molecule total methylation (A) and hydroxymethylation (B) patterns of each region (represented as one vertical blue line) were determined based on the deep loci-specific Bis/TAB-seq, respectively. Methylation patterns were separated into five groups based on the percentage of CpGs per region being methylated and/or hydroxymethylated (A) and hydroxymethylated (B). For each region (vertical blue line), the average total methylation (A) or hydroxymethylation (B) was calculated (x-axis) and the frequency of each pattern was plotted along the y-axis (summing up to 100%). Five different patterns were defined based on the proportion of methylated or hydroxymethylated CpGs of all CpGs per region (0, 0–10, 10–50, 50–80, 80–100%). [file 13072_2017_123_MOESM4_ESM.pdf]

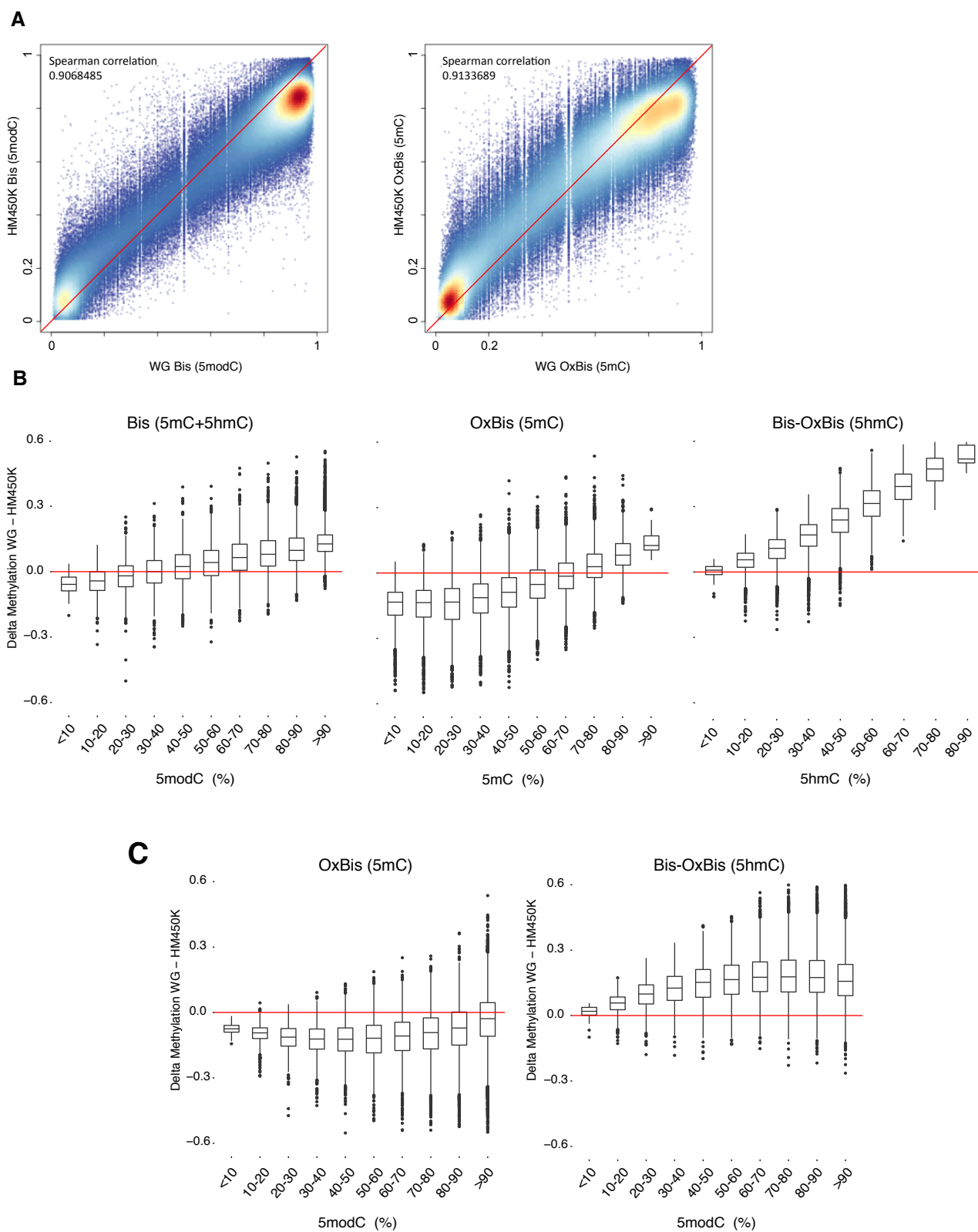

Skvortsova Supp Figure S4

Supplement: Supplementary file 5 — Additional file 5. Figure S4. WG Bis/OxBis-seq and HM450K Bis/OxBis correlation analysis. A Comparison of 5modC (Bis, left) and 5mC (OxBis, right) between WG and HM450K Bis/OxBis across CpG sites interrogated by the HM450K and having at least 10× coverage on the WG Bis/OxBis. B Boxplots showing the difference in 5modC (Bis, left), 5mC (OxBis, middle) and 5hmC (Bis-OxBis, right) between WG and HM450K Bis/OxBis at different levels of the corresponding modification. Only CpG sites (n = 42,537) considered as significantly hydroxymethylated by both approaches are included. C Boxplots showing the relationship between the total methylation levels according to the WG Bis/OxBis (x-axis) and the difference in 5mC (left) and 5hmC (right) between approaches. The difference is calculated as HM450K methylation value subtracted from the WG methylation value (y-axis). [file 13072_2017_123_MOESM5_ESM.pdf]

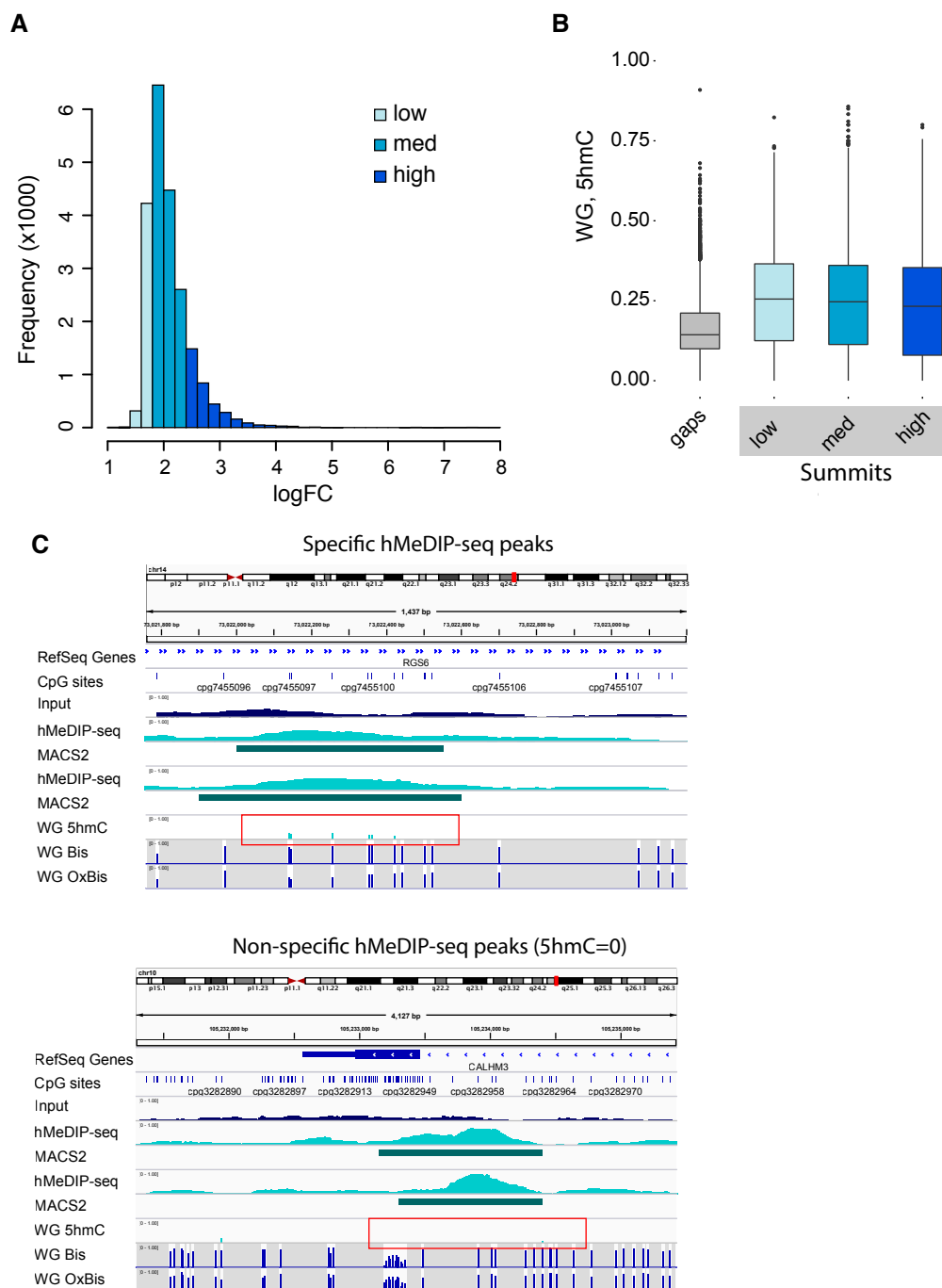

Skvortsova Supp Figure S5

Supplement: Supplementary file 6 — Additional file 6. Figure S5. Validation of the hMeDIP-seq approach with the WG Bis/OxBis-seq. A The histogram showing the distribution of the hMeDIP-seq signal normalized to the input (logFC). The colours indicate the cut-offs set to depict low (log FC < 1.8), medium (log FC > 1.8 and <2.4) and high (log FC > 2.4) hMeDIP signal enrichment. B The distribution of WG-derived 5hmC levels at hMeDIP summits expanded ±150 bp binned based on their logFC values; and gaps between them. C Screen shots of genomic regions showing specific (above) and non-specific (below) hMeDIP-seq peaks. [file 13072_2017_123_MOESM6_ESM.pdf]
