# Supplementary material for: Comprehensive evaluation of genome-wide 5-hydroxymethylcytosine profiling approaches in human DNA
Source: Epigenetics Chromatin. 2017 Apr 20;10:16. doi: 10.1186/s13072-017-0123-7 (PMC5397694; doi:10.1186/s13072-017-0123-7)
Supplement: Supplementary file 7 — Additional file 7. Figure S6. TAB-Seq amplicon validation of hMeDIP-seq in cell line DNA. Screen shots showing hMeDIP-seq peaks. WG Bis/OxBis data are shown. Grey denotes regions not covered >10× by WG Bis/OxBis. Locus-specific TAB-Seq validation showing mod C (5mC + 5hmC) and 5hmC across the amplicons with %5mC and %5hmC for each CpG site indicated. [file 13072_2017_123_MOESM7_ESM.pdf]

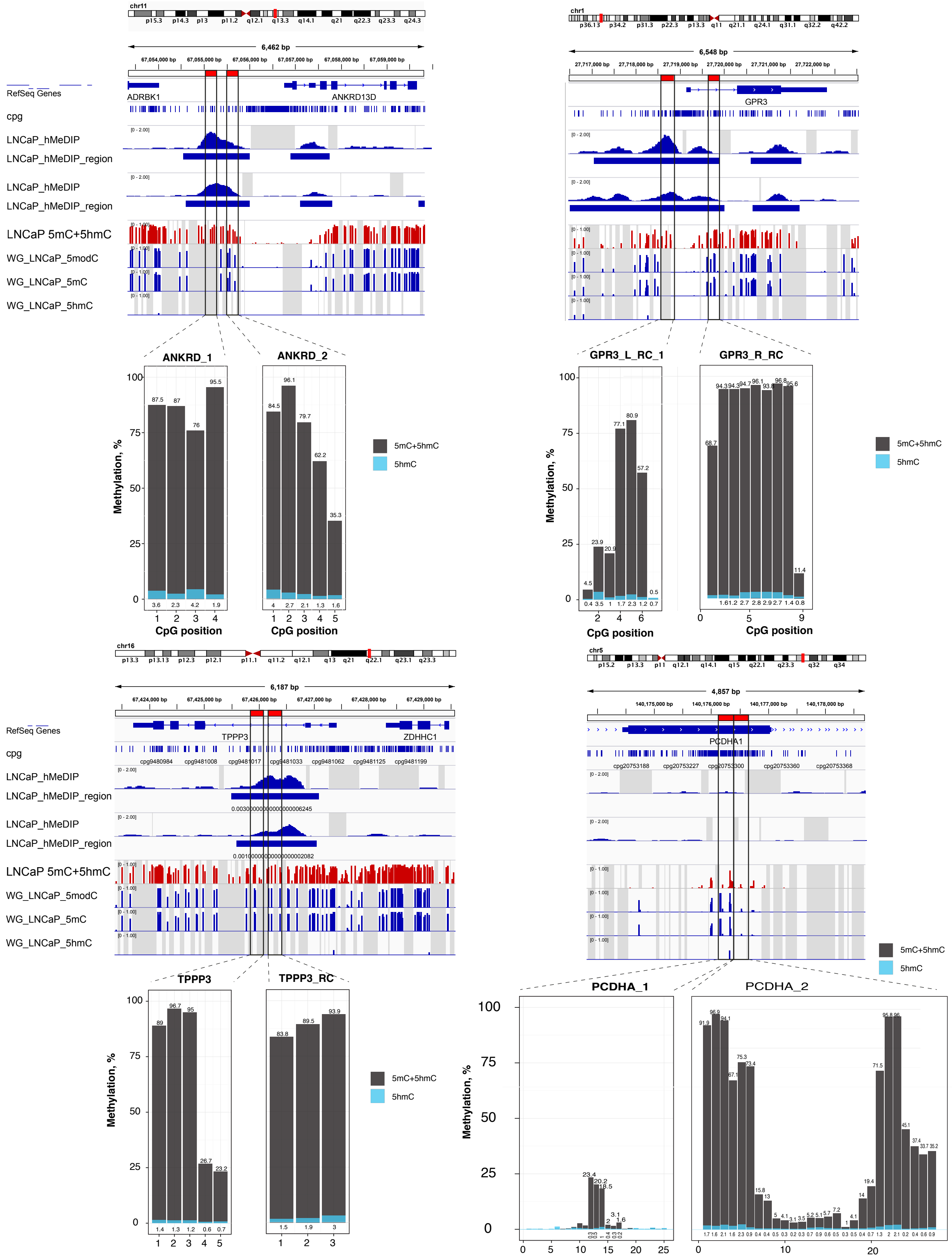

Skvortsova Supp Figure S6
